# Tissue Distribution, Excretion, and Interaction With Human Serum Albumin of Total Bioflavonoid Extract From *Selaginella doederleinii*

**DOI:** 10.3389/fphar.2022.849110

**Published:** 2022-04-29

**Authors:** Bing Chen, Dafen Xu, Zhijun Li, Yafei Jing, Luping Lin, Shaoguang Li, Liying Huang, Xiuwang Huang, Ailin Liu, Xinhua Lin, Hong Yao

**Affiliations:** ^1^ Key Laboratory of Nanomedical Technology (Education Department of Fujian Province), School of Pharmacy, Nano Medical Technology Research Institute, Fujian Medical University, Fuzhou, China; ^2^ Department of Pharmaceutical Analysis, School of Pharmacy, Fujian Medical University, Fuzhou, China; ^3^ Department of Orthopedic, The First Affiliated Hospital, Fujian Medical University, Fuzhou, China; ^4^ Department of Pharmacy, Xiamen Humanity Hospital, Fujian Medical University, Xiamen, China; ^5^ Fujian Key Laboratory of Drug Target Discovery and Structural and Functional Research, Fujian Medical University, Fuzhou, China

**Keywords:** selaginella doederleinii, bioflavonoid, tissue distribution, excretion, human serum albumin

## Abstract

*Selaginella doederleinii* Hieron is a traditional Chinese medicinal herb widely used to treat different cancers. Previously, we showed that the total bioflavonoid extract of *S. doederleinii* (TBESD) exhibits anti-carcinogenic activities both *in vitro* and *in vivo*. However, the plasma protein binding and pharmacokinetics parameters of TBESD remain unclear. To investigate plasma protein binding, tissue distribution, and excretion of TBESD, rats were administered a single dose of TBESD (600 mg/kg) intragastrically and tissue distribution and excretion of TBESD components were determined by rapid high-performance liquid chromatography and tandem mass spectrometry. TBESD binding to human serum albumin (HSA) was assessed by fluorescence spectroscopy. TBESD components amentoflavone, delicaflavone, robustaflavone, 2″,3″-dihydro-3′,3‴-biapigenin, and 3′,3‴-binaringenin were rapidly absorbed and distributed in various tissues, mostly in the lungs, kidneys, and ovaries, without long-term accumulation. The excretion of bioflavonoids occurred mostly via the intestinal tract and constituted 30% of the administered dose up to 48 h. Spectral analysis indicated that TBESD had a dynamic quenching effect on HSA by binding to one HSA site through hydrophobic interactions and hydrogen bond formation. This is the first comprehensive report on the tissue distribution, excretion, and plasma protein binding of TBESD. This study provides important information on TBESD pharmacokinetics necessary for its further development into a therapeutic form for clinical applications.

## Graphical Abstract

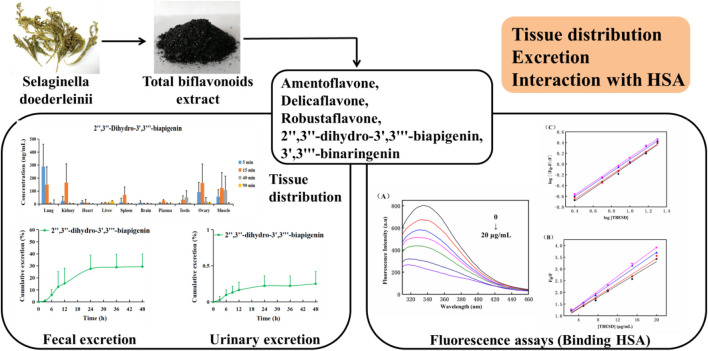

## Introduction

*Selaginella doederleinii* Hieron, a perennial herb with medicinal properties in southern China, has been traditionally used in folk medicine to treat inflammation, cardiovascular disease, and malignant tumors (Sui et al., 2016; Zou et al., 2017a; Zou et al., 2017b). The antitumor effect of *S. doederleinii* has garnered attention because its extract has been shown to considerably inhibit tumor growth *in vivo* (Yao et al., 2017; Li et al., 2020). Previous phytochemical studies have indicated that the main biologically active ingredients in *S. doederleinii* are flavonoids. Among the flavonoids, amentoflavone, delicaflavone, robustaflavone, 2″,3″-dihydro-3′,3‴-biapigenin, and 3′,3‴-binaringenin are considered to be mostly responsible for the antitumor effects of *S. doederleinii* (Chen et al., 2020; Kang et al., 2021; Liu et al., 2021). Functional pharmacological analysis revealed that the total bioflavonoid extract of *S. doederleinii* (TBESD), especially delicaflavone, can induce ROS-mediated apoptosis via caspase-dependent pathway accompanying with cell cycle arrest and inhibition of MAPK signaling cascades (Yao et al., 2019; Yao et al., 2020). In addition, delicaflavone can induce autophagy cell death by inhibiting the Akt/mTOR/p70S6K signaling pathway (Sui et al., 2016; Li et al., 2020). Overall, these studies suggest that TBESD is a promising anti-cancer candidate that deserves further investigation.

Previous pharmacokinetics studies on delicaflavone and TBESD have indicated that when administered as single oral (30–60 and 300–600 mg/kg, respectively) or intravenous (4 and 5–15 mg/kg, respectively) doses, amentoflavone, delicaflavone, robustaflavone, 2″,3″-dihydro-3′,3‴-biapigenin, and 3′,3‴-binaringenin have a short *in vivo* half-life and oral bioavailability below 3.5% (Chen et al., 2018). Analysis of tissue distribution of delicaflavone after intravenous administration has illustrated distribution predominantly in the livers, lungs, and kidneys, followed by rapid elimination. However, to the best of our knowledge, there are no specific reports on the tissue distribution, excretion, and plasma protein binding of TBESD (Chen et al., 2021).

Pharmacokinetic analysis of TBESD would reveal the absorption, distribution, metabolism, and excretion of its ingredients *in vivo* (Li et al., 2019). Furthermore, it may help elucidate their functional mechanisms, pharmacodynamics, and clinical efficacy, providing valuable information for the rational use of this herbal medicine and its further development (Zhu et al., 2020; Matsumoto et al., 2021). Therefore, pharmacokinetic investigation of TBESD is critical for understanding its safety and pharmacological and clinical effectiveness (Liao et al., 2021).

The pharmacokinetic parameters of drugs, such as absorption, metabolism, tissue distribution, and excretion, strongly depend on drug affinity to serum proteins, especially serum albumins, which are extensively used in drug-binding studies (Rabbani and Ahn, 2019). Human serum albumin (HSA), a primary protein in human blood, is known for its high drug affinity and the consequent effects on drug solubility, stability, and toxicity reduction (Cao et al., 2019; Qiu et al., 2020). Therefore, HSA is one of the main targets in the prediction of pharmacokinetic profiles of candidate therapeutic agents, and it is important to study TBESD binding to HSA in order to fully characterize the reactions of an organism to TBESD.

High-performance liquid chromatography-electrospray ionization-tandem mass spectrometry (HPLC-ESI-MS/MS) is an analytical method that has been widely applied in the pharmacokinetic analysis of multiple components in traditional Chinese medicines because of its high sensitivity and selectivity (Romański et al., 2017; Wang et al., 2021). In the present study, we analyzed TBESD tissue distribution and excretion by HPLC-ESI-MS/MS and its binding to HSA by fluorescence spectroscopy. The results provide not only reliable pharmacokinetic profiles of TBESD ingredients but also more information about the quality and functional mechanisms of TBESD.

## Methods

### Chemicals and Reagents

HPLC-grade acetonitrile and HPLC-grade methanol used in the study were purchased from Merck (Darmstadt, Germany). HPLC-grade acetic acid was purchased from Aladdin (Shanghai, China) and analytical-grade ethanol was obtained from Sinopharm Chemical Reagents (Shanghai, China). Free-fatty acid HSA (lyophilized powder) was acquired from Sigma-Aldrich (St. Louis, MO, United States). Reference standards of amentoflavone (batch number: 160,529) and chrysin (150,204) were purchased from Winherb (Shanghai, China). Delicaflavone, robustaflavone, 2″,3″-dihydro-3′,3‴-biapigenin, and 3′,3‴-binaringenin (purity ≥98%) were isolated from *S. doederleinii* and their molecular structures were fully characterized by FT-IR, UV, MS, ^1^H-NMR, and ^13^C-NMR (Chen et al., 2019). Ultrapure water was purified using a Milli-Q system (MA, United States).

### Plant Material

Dried *S. doederleinii* Hieron was obtained from a local traditional Chinese medicine store in Fuzhou, and identified by Pro. Yao (Fujian Medical University, Fuzhou, China). A voucher specimen (batch number: 201,909) has been deposited at the Phytochemistry Laboratory, Fujian Medical University (Fuzhou, China).

### Preparation of Herb Extracts

The process of obtaining TBESD and characterization of its main components were performed as previously described (Li et al., 2014). The contents of amentoflavone, delicaflavone, robustaflavone, 2″,3″-dihydro-3′,3‴-biapigenin, and 3′,3‴-binaringenin in TBESD, quantified by our previous HPLC method (Supplementary Figure S1), was 103.82, 35.12, 37.52, 44.40, and 53.36 mg/g, respectively.

### Animals

Thirty healthy Sprague–Dawley rats (weighing 250 ± 20 g) were obtained from the Laboratory Animal Center of Fujian Medical University (Fuzhou, China). All rats were maintained in an environmentally controlled breeding room (temperature: 25 ± 2°C and relative humidity: 55 ± 5%) at a 12-/12-h light/dark cycle and were observed for 1 week before the experiment. The rats were provided rat feed and water *ad libitum*; before drug administration, they were fasted for 12 h but were allowed free access to water. All experiments were conducted in accordance with the Guidelines for the Care and Use of Laboratory Animals approved by the Animal Ethics Committee of Fujian Medical University.

### HPLC-ESI-MS/MS

HPLC-ESI-MS/MS was conducted on a Shimadzu LC-20AD HPLC system with a Shimadzu LC/MS-8040 instrument (Shimadzu, Kyoto, Japan) using an Ultimate^®^ XB-C18 column (50 mm × 4.6 mm, 3.5 μm; Welch Materials, Inc., Ellicott, MD, United States). The sample injection volume was 5 μL. Gradient elution was performed at 30°C with 0.5% glacial acetic acid in water (solvent A) and acetonitrile (solvent B) at a flow rate of 0.2 ml/min. The gradient elution was set as follows, 0–1 min (40–44% B); 1–14 min (44–49.5% B); 14–15 min (49.5–95% B); 15–16 min (95% B); 16–18 min (40% B) for equilibration. The sample injection volume was 5 μL.

Triple-quadrupole MS/MS detection was carried out on a Shimadzu LC/MS-8040 system with electrospray ionization (ESI). Multiple reaction monitoring analysis in the negative ion mode was conducted based on ion transitions of amentoflavone, delicaflavone, robustaflavone, 2″,3″-dihydro-3′,3‴-biapigenin, 3′,3‴-binaringenin, and chrysin (internal standard, IS) at m/z 537.08→375.00 (CE = 35 V), *m/z* 537.08→255.00 (CE = 55 V), m/z 537.08→309.10 (CE = 40 V), *m/z* 541.11→237.09 (CE = 30 V), *m/z* 539.10→387.15 (CE = 45 V), and *m/z* 253.24 → 143.00 (CE = 28 V), respectively.^11^ The optimized MS parameters were set as follows: ion spray voltage, 6.0 kV; heat block temperature, 400°C; DL temperature, 250°C; drying gas flow, 12 L/min; nebulizer gas flow, 3 L/min. Data acquisition and processing were performed using LabSolutions LCMS Ver. 5.5 software.

### Preparation of Standard Solutions and Quality Control (QC) Samples

Standard stock solutions of amentoflavone, delicaflavone, robustaflavone, 2″,3″-dihydro-3′,3‴-biapigenin, 3′,3‴-binaringenin, and chrysin were prepared by dissolving them individually in methanol to a final concentration of 1 mg/ml; standard working and QC solutions were obtained by further dilution in methanol. For tissue distribution and excretion analyses, standard working solutions were prepared by serial dilution to obtain a linear concentration gradient. Calibration standards of delicaflavone, robustaflavone, 2″,3″-dihydro-3′,3‴-biapigenin, and 3′,3‴-binaringenin were prepared by spiking drug-free blank biological samples with the working solutions to obtain concentrations of 2, 5, 10, 25, 50, 125, 250, and 500 ng/ml and QC samples of 6, 80, and 400 ng/ml; for amentoflavone, standards of 4, 10, 20, 50, 100, 250, 500, and 1,000 ng/ml, and QC samples of 12, 160, and 800 ng/ml were prepared. The chrysin stock solution was diluted to a final concentration of 50 ng/ml. All stock solutions were stored at 4°C until use.

### Sample Preparation

To obtain tissue samples for analysis, each organ was weighed, diced into small pieces, and homogenized in three volumes of ice-cold physiological saline. Thereafter, 100 μL of the tissue homogenate was spiked with 10 μL of IS working solution, mixed by vortexing with 300 μL of methanol for 2 min, centrifuged at 15,000 × *g* for 20 min at 4°C to precipitate proteins, and the resultant supernatant (5 μL) was used for HPLC-MS/MS.

The feces were dried at 50°C to a constant weight and homogenized in three volumes of physiological saline-methanol (1:1, v/v). Thereafter, 100 μL of fecal and urine samples were processed as described above for tissue samples. The HPLC-ESI-MS/MS method developed and validated previously was only used for rat plasma samples. In the present study, the method was further verified for specificity, linearity, accuracy, precision, recovery, and stability of tissue, urine, and fecal samples.

### Tissue Distribution and Excretion

To assess the tissue distribution of orally administered TBESD, 24 Sprague–Dawley rats (12 females and 12 males, weight 200 ± 20 g) were randomly divided into four groups (3 females and 3 males per group) and intragastrically administered a single dose of TBESD (600 mg/kg) dissolved in a mixture of ethanol (2%) and propylene glycol (3.5%) in physiological saline (pH adjusted to 7.5 with 0.5 M NaOH). The rats were sacrificed by cervical dislocation at 5, 15, 40, and 90 min after TBESD administration; the time points for tissue collection were determined based on the concentration–time curves. The brain, heart, kidney, liver, lung, ovary, spleen, testes, and muscle tissues were immediately harvested, thoroughly rinsed in ice-cold physiological saline, dried with filter paper, and stored at −80°C until analysis.

To investigate urinary and fecal excretion, 3 female and 3 male rats housed in individual stainless-steel metabolic cages were orally administered a single dose of TBESD (600 mg/kg), and urine and fecal samples were collected after 0–3, 3–6, 6–9, 9–12, 12–24, 24–36, and 36–48 h. After drying the fecal samples to a constant weight at 50°C and measuring urine sample volumes, the specimens were stored at −80°C.

### Binding to HSA

The interactions of TBESD with HSA were analyzed by fluorescence spectroscopy. HSA (10 μM in phosphate buffer, pH 7.4) was incubated in the presence and absence of various TBESD concentrations (2.5, 5, 7.5, 10, and 20 μg/ml), and HSA intrinsic fluorescence spectra were recorded at 37°C. Fluorescence measurements were performed using a Cary Eclipse spectrofluorometer (CA, United States) with a thermostat bath using a 1.0-cm quartz cuvette. The width of the excitation and emission slits was set to 5 nm. An excitation wavelength of 295 nm was used in the experiments to avoid interference from tryptophan and/or tyrosine residues (Strugała et al., 2018). To avoid the inner filter effect, the fluorescence intensities were corrected using the following formula:

$$F_{corr}=\;F_{obs}\times antilog\left[\frac{\left(A_{em}+A_{ex}\right)}{2}\right]$$ (1)

where, A_em_ and A_ex_ are the absorbance of the test sample at the excitation and emission wavelengths, respectively; F_obs_ and F_corr_ are the observed and corrected fluorescence intensities, respectively.

Fluorescence quenching experiments were performed at different concentrations of TBESD (2.5, 5, 7.5, 10, and 20 μg/ml) to HSA (10 μM) at different temperatures (298, 303, 308, and 313 K). The emission spectra were recorded in the range of 300–480 nm and then calculated using the Stern–Volmer equation:

$$\frac{F_{0}}{F}=1+K_{SV}\left[Q\right]=1+\;k_{q}\tau_{0}\left[Q\right]$$ (2)

where, F and F_0_ are HSA fluorescence intensities in the presence and absence, respectively, of the quencher (TBESD); [Q] is the concentration of TBESD; K_SV_ is the dynamic quenching constant; K_q_ is the apparent bimolecular quenching rate constant; and τ_0_ is the fluorophore lifetime without TBESD (10^–8^ s).

The apparent binding constant (K_b_) and number of binding sites (n) per macromolecule at the corresponding temperature were analyzed using the modified Stern–Volmer equation:

$$\log\left\{\frac{F_{0}-F}{F}\right\}=\log K_{b}+n\log\left[Q\right]\;$$ (3)

The thermodynamic parameters of the TBESD-HSA binding were used to determine the binding mode. The thermal dependency of the binding constant was investigated at 298, 303, 308, and 313 K, and enthalpy and entropy changes (ΔH° and ΔS°, respectively) were analyzed based on the van’t Hoff plot to determine the binding forces between TBESD and HSA:

$$\ln K_{b}=-\left(\frac{\text{Δ}H{}^{\circ}}{RT}\right)+\left(\frac{\text{Δ}S{}^{\circ}}{R}\right)$$ (4)

where, R is the universal gas constant and T is the absolute temperature.

Assuming that ΔH° is nearly constant in the studied temperature range, the free energy change (ΔG°) can be calculated from the Gibbs equation:

ΔG°= ΔH°−ΔS° (5)

### Data Analysis

Data acquisition and processing were conducted using LabSolutions LCMS Ver.5.5 software. The concentrations of bioflavonoids in tissues, urine, and feces were determined using the calibration curve of each analysis batch. The pharmacokinetic parameters of bioflavonoids were calculated using a non-compartmental approach with DAS 3.0 Version (Shanghai, China).

All data are expressed as mean ± standard deviation (SD). Independent *t*-tests were performed to assess the differences between the groups; *p* < 0.05 was considered to indicate statistical significance.

## Results

### Method Validation

**FIGURE 1:**
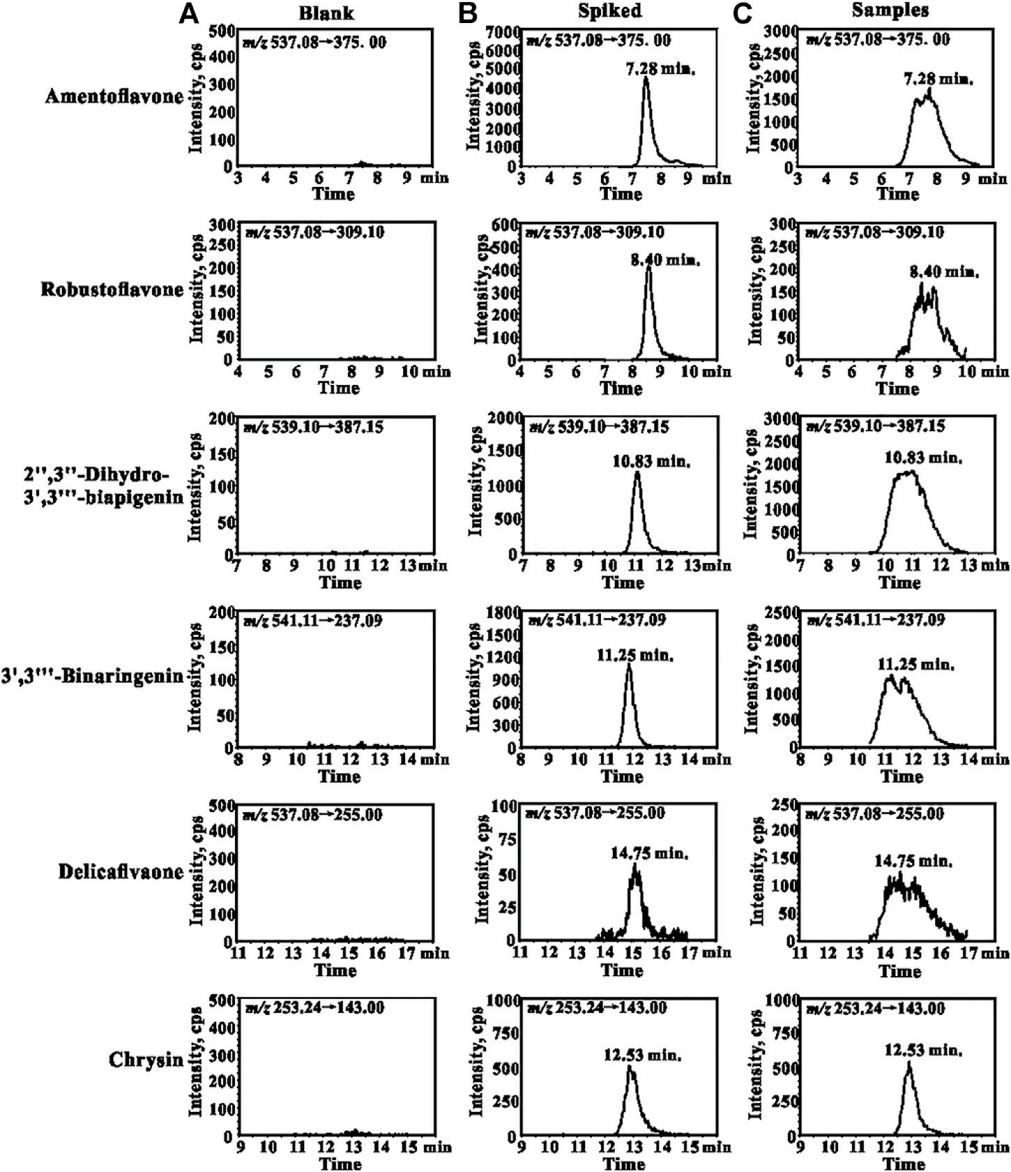
Representative MRM chromatograms for amentoflavone, robustaflavone, 2″,3″-dihydro-3′,3‴-biapigenin, 3′,3‴-binaringenin, delicaflavone, and chrysin (IS): **(A)** blank lungs sample; **(B)** blank lungs spiked with QCM and IS (50 ng/ml); **(C)** lungs sample collected from the rat 5 min after oral administration of TBESD at a dose of 600 mg/kg.

A sensitive and reliable HPLC-ESI-MS/MS method for the simultaneous quantification of five bioflavonoids (amentoflavone, delicaflavone, robustaflavone, 2″,3″-dihydro-3′,3‴-biapigenin, and 3′,3‴-binaringenin) in rat tissue, urine, and fecal samples was successfully developed and validated according to the Food and Drug Administration guidelines (Kamble et al., 2021; Shen et al., 2021). The representative chromatograms of blank rat lung homogenate, blank lung homogenate spiked with QC and IS samples, and lung homogenate samples collected 5 min after TBESD (600 mg/kg) administration are shown in Figure 1. Amentoflavone, robustaflavone, 2″,3″-dihydro-3′,3‴-biapigenin, 3′,3‴-binaringenin, IS, and delicaflavone were eluted at approximately 7.2, 8.4, 10.8, 11.2, 12.5, and 14.7 min, respectively. No detectable interfering peaks were observed in tissue, urine, or fecal samples at the retention time close to that of the five bioflavonoids and IS. The detailed method validation parameters are summarized in Tables 1–4 and Supplementary Tables S1–S12. The linearity of all calibration curves was characterized with regression coefficients (*R* ^2^) of 0.99 or higher. The intra- and inter-day precision (RSD, %) and accuracy (RE, %) of the method for each bioflavonoid were within the acceptable limit of 15%, and the matrix effect and extraction recovery of each bioflavonoid were consistent among the three different concentrations (n = 5 at high, medium, and low) and constituted >80% of the response. The five bioflavonoids were stable on the bench for up to 8 h, in the autosampler for up to 12 h, at −80°C for up to 2 months at three different concentrations, and after up to three cycles of freezing and thawing (Du et al., 2020).

**TABLE 1:** Linear regression data of biflavonoids in lungs.

Analyte	Linear Range (ng/ml)	LLOQ (ng/ml)	Regression Equation (w = 1/X^2^)
Amentoflavone	4–1,000	0.37	y = 1.261x+0.056 (*R* ^2^ = 0.999)
Robustaflavone	2–500	0.26	y = 0.218x+0.004 (*R* ^2^ = 0.999)
2″,3″-Dihydro-3′,3‴-biapigenin	2–500	0.48	y = 0.671x+0.003 (*R* ^2^ = 0.999)
3′,3‴-Binaringenin	2–500	1.29	y = 0.585x+0.010 (*R* ^2^ = 0.998)
Delicaflavone	2–500	1.63	y = 0.035x+0.021 (*R* ^2^ = 0.997)

**TABLE 2:** Method validation of amentoflavone in rat tissue homogenates (n = 5).

Sample matrix	Con	Intra-day		Inter-day	
	ng/mL	Precision (RSD, %)	Accuracy (RE, %)	Precision (RSD, %)	Accuracy (RE, %)
**Lungs**	12	2.46	3.54	3.81	0.88
	160	2.70	4.68	3.69	2.64
	800	3.18	6.68	2.88	4.65
**Liver**	12	3.45	-5.55	4.22	-1.83
	160	1.83	-0.79	2.01	1.13
	800	1.29	1.00	1.20	0.60
**Heart**	12	2.20	2.70	3.26	3.65
	160	1.68	1.25	2.09	2.34
	800	3.21	-3.02	1.52	0.55
**Spleen**	12	0.40	0.60	0.86	0.16
	160	0.66	0.06	1.53	0.82
	800	0.82	-0.42	1.34	0.06
**Kidney**	12	2.92	-1.57	2.01	-2.72
	160	1.44	-0.79	2.25	0.85
	800	0.25	0.25	0.75	-0.14
**Brain**	12	1.29	3.71	1.48	2.44
	160	1.27	-0.32	3.55	1.09
	800	3.47	1.69	1.73	2.14
**Testis**	12	4.29	2.83	2.51	4.02
	160	3.77	0.54	2.38	1.06
	800	1.58	-0.60	1.30	0.71
**Ovary**	12	3.46	1.49	2.95	2.05
	160	4.63	-0.21	4.35	-0.90
	800	1.52	1.25	1.15	-0.29
**Plasma**	12	4.08	-0.71	0.93	0.66
	160	3.59	1.39	1.63	0.46
	800	3.61	0.83	0.81	0.62
**Muscle**	12	3.18	0.95	2.57	1.49
	160	3.17	1.75	3.12	2.40
	800	6.21	-1.26	5.22	0.03

Conc., concentration. RSD, relative S.D. (calculated from S.D., divided by mean and multiplied by 100).

**TABLE 3:** Matrix effect and extraction recovery of amentoflavone in rat tissue homogenates (n = 5).

Sample matrix	Spiked con	Matrix Effect		Extraction Recovery	
	(ng/ml)	Mean ± SD (%)	RSD (%)	Mean ± SD (%)	RSD (%)
**Lungs**	12	102.56 ± 3.10	3.02	98.17 ± 2.36	2.40
	160	104.80 ± 4.08	3.90	97.57 ± 0.66	0.67
	800	101.54 ± 1.85	1.82	101.73 ± 3.10	3.05
**Liver**	12	101.38 ± 3.94	3.89	83.29 ± 8.74	10.49
	160	103.18 ± 2.38	2.30	97.94 ± 0.61	0.62
	800	98.44 ± 1.14	1.15	101.64 ± 5.03	4.95
**Heart**	12	97.29 ± 2.26	2.32	106.40 ± 6.93	6.52
	160	102.37 ± 0.33	0.33	98.84 ± 2.27	2.29
	800	101.24 ± 0.30	0.30	96.16 ± 1.55	1.62
**Spleen**	12	98.67 ± 1.87	1.89	99.09 ± 6.52	6.58
	160	101.97 ± 3.19	3.13	98.61 ± 3.74	3.79
	800	97.93 ± 1.25	1.27	100.98 ± 0.30	0.30
**Kidney**	12	101.50 ± 5.55	5.47	93.13 ± 6.32	6.79
	160	99.45 ± 3.40	3.42	102.63 ± 5.00	4.88
	800	93.00 ± 1.78	1.91	103.79 ± 2.09	2.01
**Brain**	12	100.41 ± 3.80	3.78	98.00 ± 1.96	2.00
	160	103.11 ± 1.84	1.78	95.56 ± 1.20	1.25
	800	98.37 ± 1.86	1.89	101.09 ± 3.16	3.13
**Testis**	12	99.92 ± 3.88	3.88	99.10 ± 4.05	4.09
	160	103.17 ± 5.11	4.95	98.18 ± 1.47	1.50
	800	99.36 ± 1.09	1.10	98.82 ± 3.23	3.27
**Ovary**	12	98.85 ± 2.12	2.14	94.09 ± 6.69	7.11
	160	103.17 ± 0.91	0.88	97.04 ± 0.73	0.75
	800	96.70 ± 1.36	1.41	100.26 ± 3.21	3.20
**Plasma**	12	100.41 ± 0.93	0.93	96.49 ± 1.53	0.02
	160	100.65 ± 1.84	1.83	100.02 ± 0.13	0.01
	800	101.61 ± 0.91	0.90	96.55 ± 1.36	0.01
**Muscle**	12	95.60 ± 2.29	2.39	103.98 ± 7.36	7.08
	160	100.45 ± 2.62	2.61	101.80 ± 8.61	8.45
	800	100.12 ± 2.53	2.53	101.71 ± 6.44	6.33

**TABLE 4:** The stability of amentoflavone in rat tissue homogenates (n = 5).

Sample matrix	Spiked con	Bench-top stability (37°C, 8 h)		Short-term stability (4°C, 12 h)		Freeze-thaw stability (Three cycles)		Long-term stability (−80°C, 60 days)	
	(ng/ml)	Bias (%)	RSD (%)	Bias (%)	RSD (%)	Bias (%)	RSD (%)	Bias (%)	RSD (%)
**Lungs**	12	2.19	7.74	4.32	5.93	0.40	3.86	4.79	4.21
	160	2.06	3.12	3.32	1.75	0.74	5.17	0.30	1.60
	800	−0.35	2.07	0.68	4.54	1.28	2.22	2.60	2.24
**Liver**	12	1.28	2.78	4.33	3.64	−1.13	2.95	−2.41	1.93
	160	−0.66	3.82	−1.86	2.96	−1.92	2.24	0.74	1.39
	800	−1.86	1.25	0.75	0.64	0.67	1.49	−1.13	2.04
**Heart**	12	0.27	2.45	−0.29	1.16	0.32	1.95	−4.76	3.53
	160	2.11	2.10	0.03	1.97	2.13	3.01	−1.35	2.86
	800	1.56	3.60	2.42	2.94	−4.90	3.35	−1.52	4.06
**Spleen**	12	−0.53	1.85	3.35	4.28	−2.27	3.98	4.04	2.19
	160	3.56	2.00	2.80	3.22	−4.11	1.82	2.02	1.99
	800	0.78	1.85	−1.09	3.25	−4.41	6.84	3.80	2.46
**Kidney**	12	−1.53	3.12	2.88	3.21	−4.01	5.30	−5.62	3.63
	160	2.02	1.01	2.39	1.42	−3.13	2.03	−3.88	1.47
	800	−2.50	4.56	−0.50	3.76	−4.85	2.39	−2.47	3.12
**Brain**	12	−0.37	1.08	1.42	3.83	0.96	2.04	4.09	2.81
	160	3.01	1.16	−3.82	4.38	2.87	2.74	−2.14	3.08
	800	−1.54	3.45	−2.72	5.21	-0.50	2.64	−0.55	2.06
**Testis**	12	0.23	1.78	−1.25	7.18	2.92	2.85	2.24	2.35
	160	1.00	0.99	1.52	2.18	1.40	1.43	2.34	2.31
	800	−2.24	1.10	−0.85	1.94	0.91	2.52	−0.63	2.10
**Ovary**	12	1.87	4.26	3.64	3.36	−0.93	6.65	2.89	1.72
	160	−0.52	2.99	2.48	2.91	3.71	6.85	0.52	1.51
	800	−0.79	2.44	1.61	1.40	1.81	2.47	−0.40	4.87
**Plasma**	12	3.58	1.73	0.26	1.55	−0.02	2.97	3.81	2.56
	160	−0.56	4.76	1.05	2.58	−0.93	4.25	0.70	3.98
	800	0.59	0.59	0.12	2.75	−2.13	3.33	−1.20	1.96
**Muscle**	12	0.03	0.78	−0.32	4.90	−0.01	3.18	2.01	1.27
	160	2.26	4.25	−0.81	3.98	−6.18	2.30	−2.14	3.59
	800	0.13	3.03	−0.22	3.42	2.02	2.90	0.76	3.74

### Tissue Distribution

**FIGURE 2:**
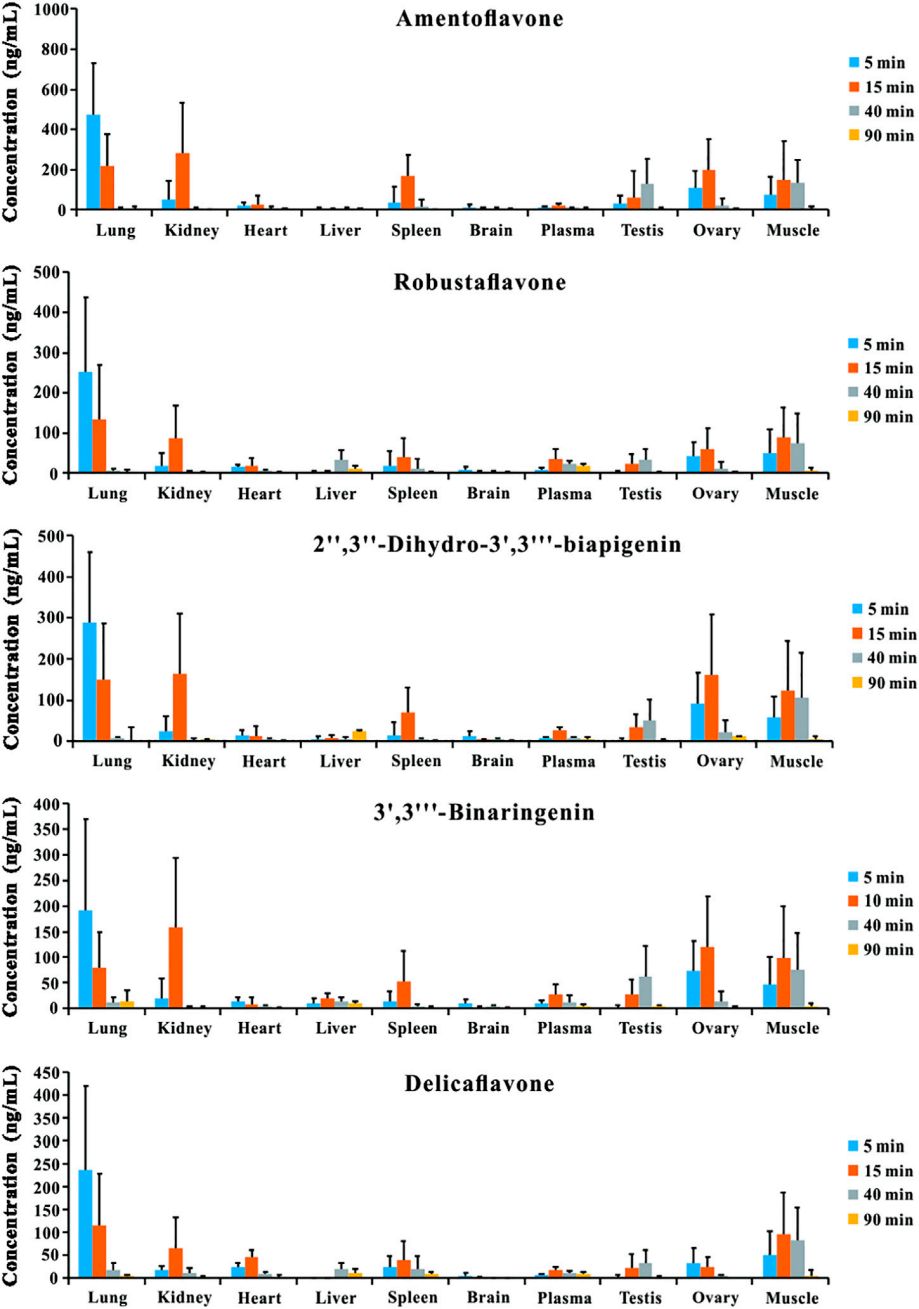
Tissue distribution profiles of amentoflavone, robustaflavone, 2″,3″-dihydro-3′,3‴-biapigenin, 3′,3‴-binaringenin, and delicaflavone in rat after oral administration of TBESD at a dose of 600 mg/kg (Mean ± SD, n = 6).

The tissue distribution profiles of the five bioactive ingredients in the rats after the oral administration of TBESD (600 mg/kg) are depicted in Figure 2. The highest concentration of the five bioflavonoids was observed in the lungs at 5 min, followed by the kidneys, muscle, ovary, spleen, and testes, suggesting that amentoflavone, delicaflavone, robustaflavone, 2″,3″-dihydro-3′,3‴-biapigenin, and 3′,3‴-binaringenin distributed rapidly in various tissues. The peak levels in the lungs, kidneys, muscle, ovary, spleen, and testes were significantly higher than those in the plasma. However, the content of the five bioflavonoids in the lungs significantly decreased, whereas that in the kidneys, ovary, and spleen initially increased and then significantly decreased. Only a small amount of the five bioflavonoids was detected in the brain, indicating that amentoflavone, delicaflavone, robustaflavone, 2″,3″-dihydro-3′,3‴-biapigenin, and 3′,3‴-binaringenin could not easily cross the blood–brain barrier. The highest content of the five bioflavonoids in the lungs after oral TBESD administration suggests that TBESD can be used to treat lung-related diseases (Sui et al., 2017). Clearly, the pharmacological effects of TBESD are results from multi-ingredients and multi-targets synergistic integration, thus, the integrated pharmacokinetic studies have carried out to reveal the overall *in vivo* process of TBESD. The half maximal inhibitory concentration (IC_50_) of an ingredient against human lung cancer cell line (take A549 cell line for example) curve was used to the integrated pharmacokinetic studies of TBESD (Shi et al., 2018). The IC_50_ of amentoflavone, delicaflavone, robustaflavone, 2″,3″-dihydro-3′,3‴-biapigenin, and 3′,3‴-binaringenin were 36.3, 13.2, 50.0, 40.2, and 19.3 μg/ml, respectively (Li et al., 2014; Sui et al., 2016). The tissue distribution profiles of multiple ingredients of TBESD are shown in Supplementary Figure S2. The weighting coefficient and integrated concentrations are calculated by following equations:

$$\omega j=\frac{1/IC_{50j}}{\sum_{1}^{n}1/IC_{50n}}$$ (6)

∑1n1IC50n=1IC501+1IC502+1IC503+⋯+1IC50n (7)

$$C_{T}=\omega_{1}C_{1}+\omega_{2}C_{2}+\omega_{3}C_{3}+\cdots +\omega_{n}C_{n}$$ (8)

Where *j* represents ingredient *j*, *ω*_*j*_ represents the weighting coefficient of the ingredient *j*, n represents the number of ingredients studied, *C*_*n*_ represents the concentration of each ingredients at time point T, and *C*_*T*_ represented the integrated concentration, respectively.

### Excretion Analysis

**FIGURE 3:**
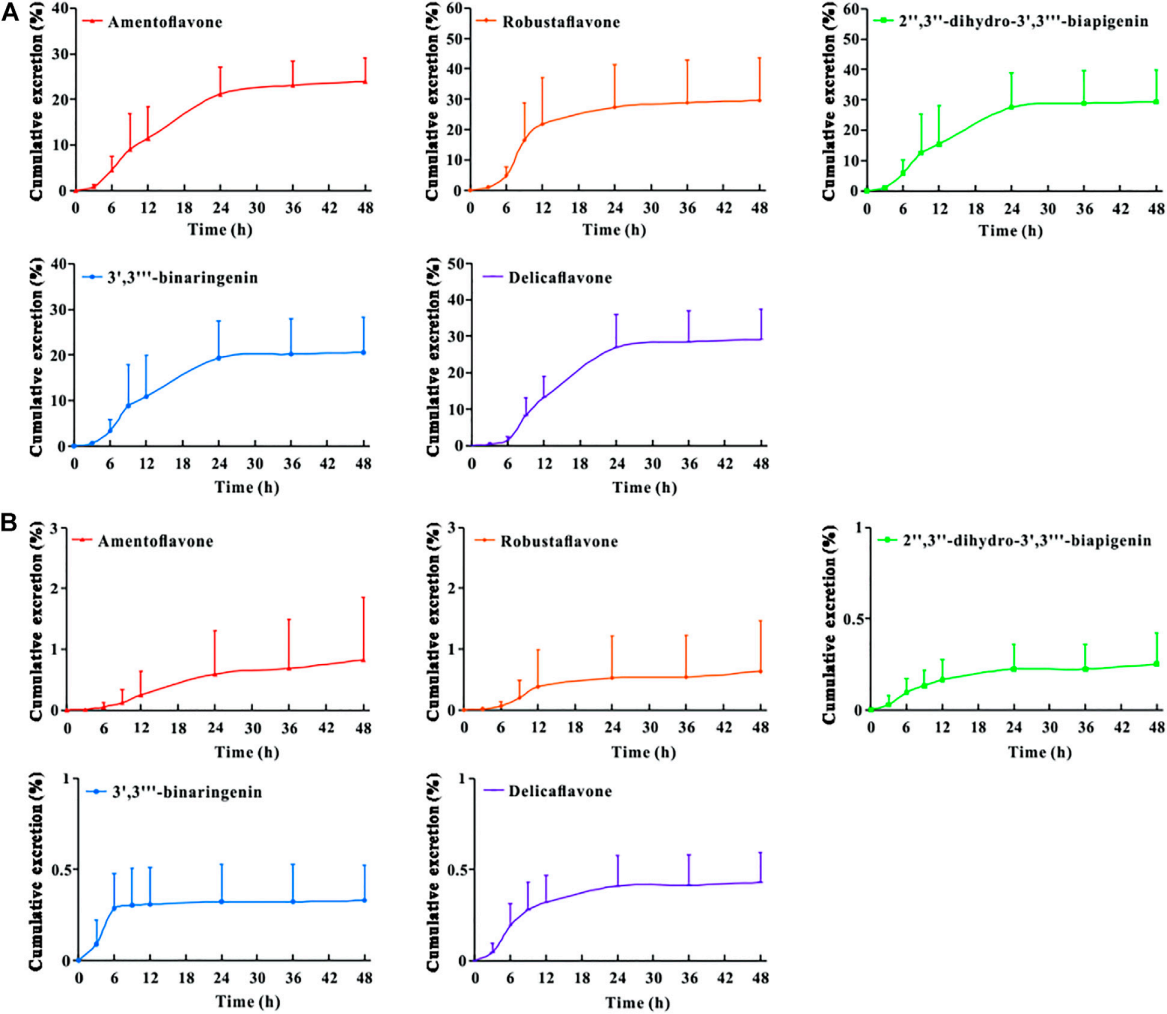
Cumulative excretion-time profiles of amentoflavone, robustaflavone, 2″,3″-dihydro-3′,3‴-biapigenin, 3′,3‴-binaringenin, and delicaflavone in rat feces **(A)** and urine **(B)** after oral administration of TBESD at a dose of 600 mg/kg (Mean ± SD, n = 6).

The urinary and fecal excretion profiles of the five bioflavonoids after a single oral administration of TBESD are shown in Figure 3. The cumulative concentration of amentoflavone, delicaflavone, robustaflavone, 2″,3″-dihydro-3′,3‴-biapigenin, and 3′,3‴-binaringenin excreted in feces and urine was 23.93 and 0.82%, 29.11 and 0.43%, 29.58 and 0.63%, 29.31 and 0.25%, and 20.51 and 0.32%, respectively, up to 48 h, indicating a considerably higher excretion rate in feces than in urine. These results suggest fecal excretion as the main excretion route for the five bioflavonoids of TBESD, and this is consistent with the findings of a previous study on flavonoids (Zheng et al., 2019).

To the best of our knowledge, this is the first study to report that TBESD is excreted mainly through feces. In addition, the cumulative excretion of the five bioflavonoids in feces after the oral administration of TBESD was considerable, which may have contributed to their low bioavailability and could be attributed to the abundant expression of organic anion transporters and multidrug resistance-related proteins in the membrane of intestinal epithelial cells (Barreca et al., 2017).

### Binding to HSA and Characterization of the Bioflavonoid-HSA Complex

**FIGURE 4:**
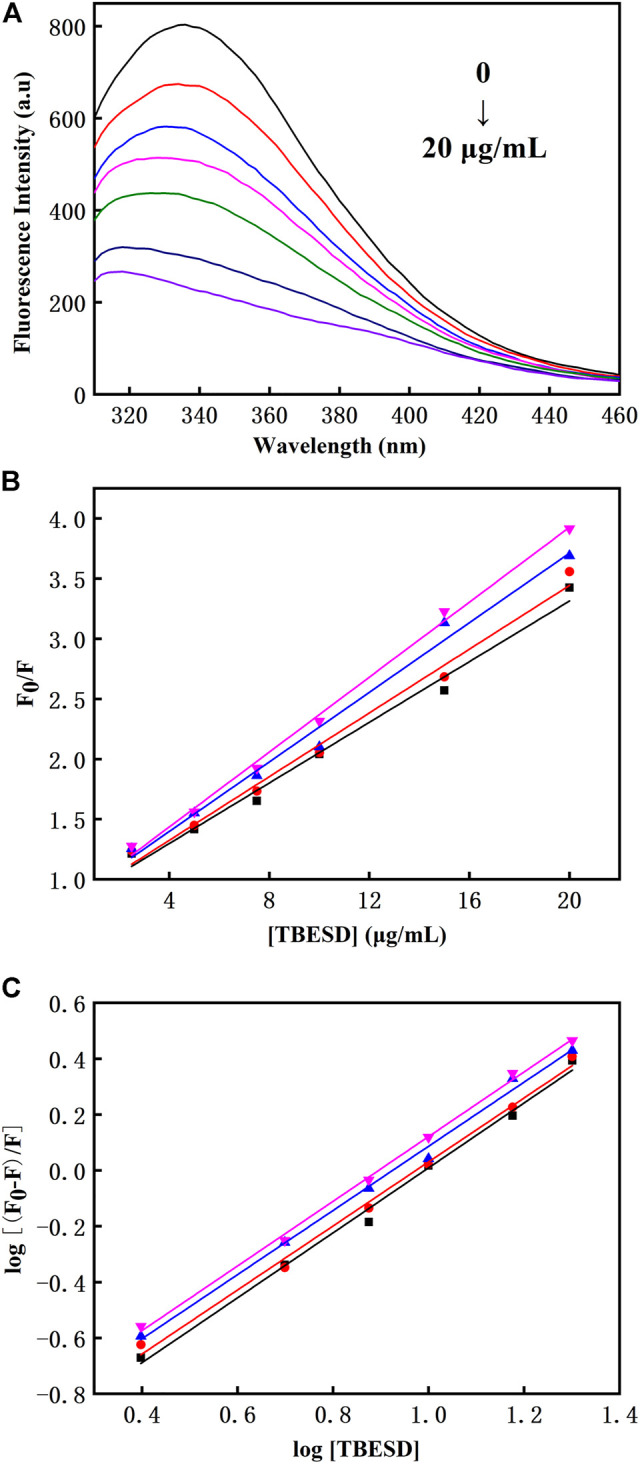
**(A)** The intrinsic fluorescence spectra of HSA (10 μM) in the absence and presence of various concentrations of TBESD (2.5, 5, 7.5, 10, and 20 μg/ml); **(B)** The Stern-Volmer plots and **(C)** the modified Stern-Volmer plots for fluorescence quenching of HSA (10 μM) by TBESD at 298 (■), 303 (●), 308 (▲), and 313 (▼) K.

The tryptophan residues in HSA are highly sensitive to polarity changes, making fluorescence spectroscopy a valuable method for studying HSA conformational shifts after binding to different ligands (Balaei and Ghobadi, 2019). TBESD exhibited very low fluorescence under the present experimental conditions. Quenching of intrinsic HSA fluorescence was measured in the presence and absence of different concentrations of TBESD at 298 K (Figure 4A). The results indicated that the fluorescence intensity of HSA decreased with the increase in TBESD concentration, suggesting the presence of tryptophan residues at or near the HSA-TBESD binding site.

There are two types of fluorescence quenching mechanisms: dynamic and static. The dynamic quenching occurs in the presence of high quencher concentrations, and the effective number of collisions increases at higher temperatures; therefore, the increase in temperature should cause an increase in the quenching constant (K_SV_). In contrast, in static quenching, the stability of the formed complex decreases with the increase in temperature, which results in the decrease in K_SV_ (Strugała et al., 2021). To elucidate the mechanism underlying TBESD quenching of HSA fluorescence, we analyzed HSA fluorescence at four different temperatures (Figure 4 and Table 5). The Stern–Volmer plots for the TBESD-HSA complexes were linear, showing a directly proportional relationship between the examined concentration range and the temperature. The number of binding sites (n) for TBESD in HSA at different temperatures is shown in Table 5. The results indicated that there was one binding site, that is, only one interaction point between the TBESD components and HSA. According to the obtained results, the most probable mechanism of HSA fluorescence quenching by TBESD should be dynamic quenching, in which higher temperatures increase the stability of the TBESD-HSA complex as indicated by higher K_SV_ and K_b_ values.

**TABLE 5:** The Stern-Volmer quenching constant (K_SV_), quenching rate constant (Kq), binding constant (K_b_) and number of binding site (n) of the interaction between HSA and TBESD at different temperatures (Mean ± SD, n = 3).

T (K)	K_SV_ × 10^4^ (ml g^−1^)	K_q_ × 10^12^ (ml g^−1^ s^−1^)	K_b_ × 10^4^ (ml g^−1^)	n
298	12.60 ± 0.60	12.60 ± 0.60	7.02 ± 0.26	1.16
303	13.24 ± 0.11	13.24 ± 0.11	7.65 ± 0.05	1.15
308	14.47 ± 0.28	14.47 ± 0.28	8.66 ± 0.17	1.15
313	15.57 ± 0.26	15.57 ± 0.26	9.18 ± 0.07	1.16

To characterize the energy changes during the interaction between TBESD and HSA, the thermodynamic binding parameters ΔH°, ΔS°, and ΔG° were calculated from the van’t Hoff plot and Gibbs equation (Table 6). The ΔH° and ΔS° values were 29.10 ± 2.86 kJ (g ml-1)^−1^ and 0.27 ± 0.01 J (g mL-1 K)^−1^, respectively. The high negative ΔG° value accompanied by positive ΔS° is indicative of a spontaneous process. The positive ΔS° value signifies hydrophobic interactions, whereas the variations in ΔH° indicate that the binding process is enthalpy-driven and probably depends on the formation of hydrogen bonds between the TBESD components and HSA. Thus, thermodynamic analysis of the binding between TBESD and HSA revealed the role of hydrophobic interactions and hydrogen bonds in complex formation.

**TABLE 6:** Thermodynamic parameters of the interaction between HSA and TBESD at different temperatures (Mean ± SD, n = 3).

T (K)	ΔS^o^ (J mol^−1^ K^−1^)	ΔH^o^ (kJ mol^−1^)	ΔG^o^ (kJ mol^−1^)
298	0.27 ± 0.01	29.10 ± 2.86	−51.26 ± 0.13
303			−52.61 ± 0.09
308			−53.96 ± 0.05
313			−55.30 ± 0.03

## Disscusion

Multiple studies have established that TBESD with an excellent antitumor effect and low toxicity *in vivo*, and deserved to be further developed as a novel anti-cancer agent (Wang et al., 2019; Xu et al., 2021). An understanding of the pharmacokinetic properties of TBESD is an essential prerequisite to the drug discovery and preclinical development. Especially pharmacokinetic studies of natural products, since they typically involve the administration of complex mixtures of substances. In this study, we revealed how the body affects the five biological activity of TBESD after administration through the mechanisms of absorption and distribution, and the effects and routes of excretion.

As our previous reported, five bioflavonoids were retained in the intestinal tract for a long time and maintained lower levels plasma concentration after a single dose oral administration of TBESD (Chen et al., 2018). In the tissue distribution study, the content of amentoflavone, delicaflavone, robustaflavone, 2″,3″-dihydro-3′,3‴-biapigenin, 3′,3‴-binaringenin and integrated TBESD in the lungs was the highest among these organs, followed by kindeys, ovary, and spleen, suggesting that the application of TBESD in the treatment of lungs, kindeys, and ovary related diseases. However, only a small amount of five bioflavonoids was detected in the brain, indicating that most flavonoids from TBESD had difficulty passing the blood-brain barrier.

To investigate the elimination of TBESD in rats, 5 major bioflavonoids were measured in the fecal and urine samples within 48 h after a single dose oral administration. The cumulative amount of amentoflavone, delicaflavone, robustaflavone, 2″,3″-dihydro-3′,3‴-biapigenin, and 3′,3‴-binaringenin was detected mainly in feces with 23.93, 29.11, 29.58, 29.31 and 20.51%, respectively, while the ratios were 0.82, 0.43, 0.63, 0.25%, and 0.32 in urine. Fecal excretion was proven to be the main excretion route for the TBESD, which may account for their poor bioavailability and membrane transporters (Čvorović et al., 2018). In addition, the absorbed flavonoids of TBESD undergo further metabolism of glucuronide, sulfation, and methylation in the epithelium of intestine and liver (Zheng et al., 2019). From the above results, the high excretion rate may be related to its incomplete absorption and metabolic way under the oral administration route, which we will share the research advances in more detail in an upcoming installment.

Fluorescence spectroscopy studies were conducted to investigate the interaction between TBESD and HSA. It is recognized that the bioavailability of many active compounds is related to the interaction and successful binding with HSA (Poureshghi et al., 2017). Forming stable binding complexes could be considered a suitable model for gaining various information of protein binding (Wang et al., 2015). Our previous study verified that the fluorescence intensity of HSA decreased regularly with increasing active ingredient concentration, due to an increased hydrophobicity of the region surrounding the tryptophan amino acid residue after binding with TBESD’s component. In the present study, the binding constant of the whole ingredients of TBESD to HSA was determined by the intrinsic fluorescence quenching of HSA. These results indicate that the strong and spontaneous binding of the whole ingredients of TBESD to HSA is responsible for poor pharmacokinetic profiles.

## Conclusion

We developed a sensitive and reliable LC-MS/MS method to simultaneously determine the concentration of five main TBESD bioflavonoids, amentoflavone, robustaflavone, 2″,3″-dihydro-3′,3‴-biapigenin, 3′,3‴-binaringenin, and delicaflavone, in rat tissues, urine, and feces after the oral administration of TBESD. We successfully applied the method to assess TBESD tissue distribution and excretion in rats. The concentration of the five bioflavonoids was the highest in the lungs, and they were mostly excreted through the feces. TBESD had a dynamic quenching effect on HSA by binding to a single HSA site through hydrophobic interactions and hydrogen bond formation. To the best of our knowledge, this is the first comprehensive study on the tissue distribution and excretion of TBESD components in rats and their interaction with HSA. Further research on the pharmacokinetics and pharmacodynamics of TBESD is needed to provide a solid foundation for the application of TBESD in clinical practice.

## Data Availability

The original contributions presented in the study are included in the article/Supplementary Materials, further inquiries can be directed to the corresponding authors.
