# Supplementary material for: Tissue Distribution, Excretion, and Interaction With Human Serum Albumin of Total Bioflavonoid Extract From Selaginella doederleinii
Source: Front Pharmacol. 2022 Apr 29;13:849110. doi: 10.3389/fphar.2022.849110 (PMC9099209; doi:10.3389/fphar.2022.849110)
Supplement: Supplementary file 1 [file DataSheet1.docx]

**Supplementary Material**

**Tissue distribution, excretion, and interaction with human serum albumin of total bioflavonoid extract from *Selaginella doederleinii***

Bing Chen^1,2†^, Dafen Xu^1,2†^, Zhijun Li^3^, Yafei Jing^2^, Luping Lin^2^, Shaoguang Li^2^, Liying Huang^2^, Xiuwang Huang^4^, Ailin Liu^2*^, Xinhua Lin^1,2*^, and Hong Yao^2,5^*

^1^Key Laboratory of Nanomedical Technology (Education Department of Fujian Province), School of Pharmacy, Nano Medical Technology Research Institute, Fujian Medical University, Fuzhou, China

^2^Department of Pharmaceutical Analysis, School of Pharmacy, Fujian Medical University, Fuzhou, China

^3^Department of Orthopedic, The First Affiliated Hospital, Fujian Medical University, Fuzhou, China

^4^Department of Pharmacy, Xiamen Humanity Hospital, Fujian Medical University, Xiamen, China

^5^Fujian Key Laboratory of Drug Target Discovery and Structural and Functional Research, Fujian Medical University, Fuzhou, China

*** Correspondence:**

Hong Yao

yauhung@126.com;

Xinhua Lin

13906909638@163.com;

Ailin Liu

ailinliu@fjmu.edu.cn

**^†^**These authors have contributed equally to this work and share first authorship

**List of Supplementary Material Captions**

**Figure. S1** HPLC chromatogram of the total bioﬂavonoids extract from *S. doederleinii*. (1) amentoflavone, (2) robustaflavone, (3) 2'',3''-dihydro-3',3'''-biapigenin, (4) 3',3'''-binaringenin and (5) delicaflavone, (6) 2,3-Dihydrohinokiflavone, (7) Chrysocauloflavone I, (8) Heveaflavone, (9) 7,4',7'',4'''-tetra-O-methylamentoflavone.

**Figure. S2** Tissue distribution profiles of integrated TBESD in rat after oral administration of TBESD at a dose of 600 mg/kg (Mean ± SD, n=6).

**Table S1** Method validation of robustflavone in rat tissue homogenates (n=5).

**Table S2** Method validation of 2′′,3′′-dihydro-3,3′′′-biapigenin in rat tissue homogenates (n=5).

**Table S3** Method validation of 3,3′′′-binaringenin in rat tissue homogenates (n=5).

**Table S4** Method validation of delicaflavone in rat tissue homogenates (n=5).

**Table S5** Matrix effect and extraction recovery of robustflavone in rat tissue homogenates (n=5).

**Table S6** Matrix effect and extraction recovery of 2′′,3′′-dihydro-3,3′′′-biapigenin in rat tissue homogenates (n=5).

**Table S7** Matrix effect and extraction recovery of 3,3′′′-binaringenin in rat tissue homogenates (n=5).

**Table S8** Matrix effect and extraction recovery of delicaflavone in rat tissue homogenates (n=5).

**Table S9** The stability of robustflavone in rat tissue homogenates (n=5).

**Table S10** The stability of 2′′,3′′-dihydro-3,3′′′-biapigenin in rat tissue homogenates (n=5).

**Table S11** The stability of 3,3′′′-binaringenin in rat tissue homogenates (n=5).

**Table S12** The stability of delicaflavone in rat tissue homogenates (n=5).

**
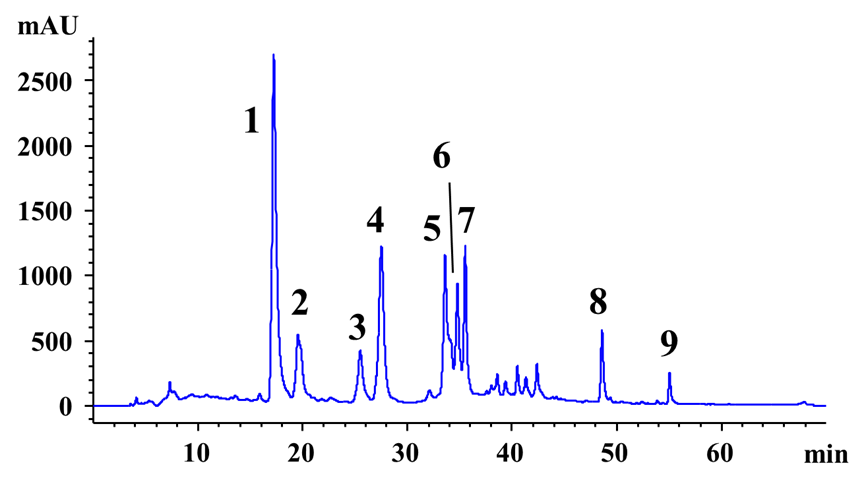
**

**Figure. S1** HPLC chromatogram of the total bioﬂavonoids extract from *S. doederleinii*. (1) amentoflavone, (2) robustaflavone, (3) 2'',3''-dihydro-3',3'''-biapigenin, (4) 3',3'''-binaringenin and (5) delicaflavone, (6) 2,3-Dihydrohinokiflavone, (7) Chrysocauloflavone I, (8) Heveaflavone, (9) 7,4',7'',4'''-tetra-O-methylamentoflavone.


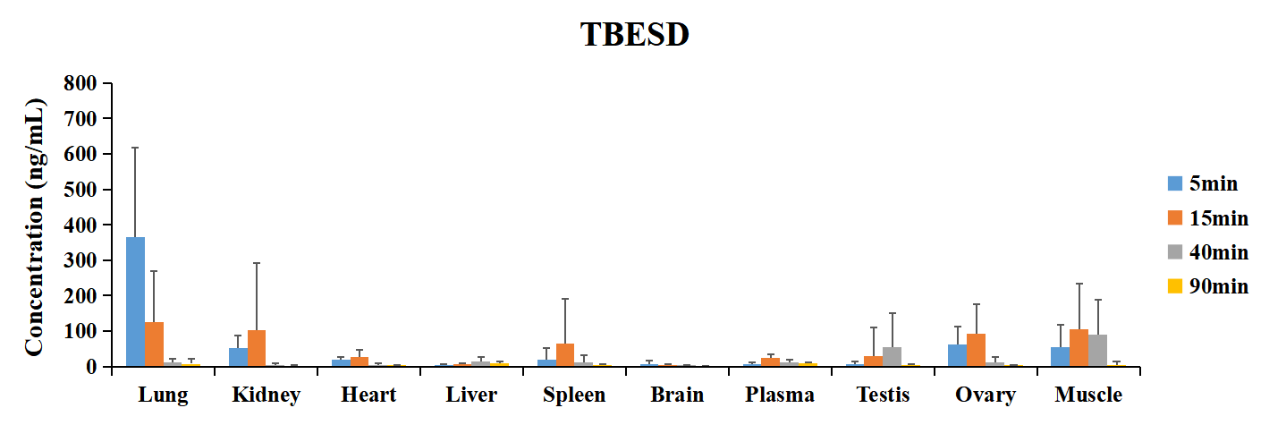


**Figure. S2** Tissue distribution profiles of integrated TBESD in rat after oral administration of TBESD at a dose of 600 mg/kg (Mean ± SD, n=6).

**Table S1** Method validation of robustflavone in rat tissue homogenates (n=5).

| **Sample matrix** | **Con.** | **Intra-day** | | **Inter-day** | |
| --- | --- | --- | --- | --- | --- |
|  | **ng/mL** | **Precision** | **Accuracy** | **Precision** | **Accuracy** |
|  |  | **(RSD, %)** | **(RE, %)** | **(RSD, %)** | **(RE, %)** |
| **Lungs** | 12 | 5.18 | 4.55 | 4.67 | -1.58 |
|  | 160 | 3.26 | 5.83 | 5.1 | 3.39 |
|  | 800 | 5.65 | 9.74 | 3.24 | 3.59 |
| **Liver** | 12 | 7.85 | 5.70 | 2.93 | 7.65 |
|  | 160 | 6.46 | 1.92 | 4.37 | 2.59 |
|  | 800 | 2.96 | 1.65 | 1.65 | 2.02 |
| **Heart** | 12 | 4.02 | -0.49 | 4.54 | 2.91 |
|  | 160 | 4.86 | 0.47 | 2.81 | 1,92 |
|  | 800 | 5.23 | -1.55 | 1.84 | 1.81 |
| **Spleen** | 12 | 4.91 | -7.47 | 4.86 | -6.26 |
|  | 160 | 3.04 | -2.64 | 1.91 | 0.46 |
|  | 800 | 5.96 | -1.02 | 2.42 | 3.93 |
| **Kidney** | 12 | 7.84 | -1.87 | 7.33 | -4.54 |
|  | 160 | 11.17 | -6.61 | 4.39 | -0.83 |
|  | 800 | 1.31 | -1.42 | 2.05 | 0.52 |
| **Brain** | 12 | 2.09 | -8.26 | 2.27 | -6.30 |
|  | 160 | 5.15 | -4.94 | 4.60 | -4.51 |
|  | 800 | 2.06 | -0.18 | 2.81 | 1.78 |
| **Testis** | 12 | 4.20 | 4.76 | 2.97 | 2.24 |
|  | 160 | 3.21 | 1.83 | 3.36 | 1.40 |
|  | 800 | 9.87 | -2.64 | 9.83 | 1.75 |
| **Ovary** | 12 | 5.85 | 1.46 | 4.38 | 3.64 |
|  | 160 | 5.13 | -0.16 | 4.87 | 2.69 |
|  | 800 | 5.04 | -0.45 | 3.55 | 3.31 |
| **Plasma** | 12 | 8.06 | -0.06 | 3.56 | 5.06 |
|  | 160 | 4.27 | 2.50 | 4.22 | -0.41 |
|  | 800 | 6.42 | 2.39 | 2.38 | 2.63 |
| **Muscle** | 12 | 4.49 | 1.80 | 7.75 | -3.37 |
|  | 160 | 5.09 | 1.55 | 4.58 | 4.60 |
|  | 800 | 3.85 | 0.54 | 2.57 | 1.82 |

**Table S2** Method validation of 2′′,3′′-dihydro-3,3′′′-biapigenin in rat tissue homogenates (n=5).

| **Sample matrix** | **Con.** | **Intra-day** | | **Inter-day** | |
| --- | --- | --- | --- | --- | --- |
|  | **ng/mL** | **Precision** | **Accuracy** | **Precision** | **Accuracy** |
|  |  | **(RSD, %)** | **(RE, %)** | **(RSD, %)** | **(RE, %)** |
| **Lungs** | 12 | 2.61 | 4.41 | 2.23 | 1.51 |
|  | 160 | 3.71 | 6.25 | 2.29 | 1.74 |
|  | 800 | 2.20 | 5.20 | 2.05 | 2.67 |
| **Liver** | 12 | 3.61 | -9.75 | 3.27 | -12.52 |
|  | 160 | 2.74 | -7.82 | 5.20 | -4.91 |
|  | 800 | 3.89 | -4.25 | 4.34 | -1.19 |
| **Heart** | 12 | 9.72 | -4.44 | 2.54 | 6.19 |
|  | 160 | 3.78 | 2.48 | 1.36 | -0.94 |
|  | 800 | 2.23 | 1.87 | 1.36 | -0.27 |
| **Spleen** | 12 | 9.09 | -1.30 | 2.60 | -1.54 |
|  | 160 | 6.61 | -4.60 | 1.28 | 2.16 |
|  | 800 | 10.38 | 0.24 | 2.61 | 5.23 |
| **Kidney** | 12 | 2.46 | -7.15 | 5.32 | -4.84 |
|  | 160 | 3.62 | -9.95 | 2.96 | -6.92 |
|  | 800 | 2.88 | -4.63 | 1.03 | -1.93 |
| **Brain** | 12 | 4.16 | -6.28 | 6.55 | -1.85 |
|  | 160 | 3.63 | -4.31 | 3.54 | -1.29 |
|  | 800 | 2.27 | 2.42 | 3.11 | -0.25 |
| **Testis** | 12 | 8.29 | 3.05 | 6.54 | 3.66 |
|  | 160 | 3.68 | -1.20 | 3.56 | 2.66 |
|  | 800 | 5.01 | -4.84 | 2.46 | 0.65 |
| **Ovary** | 12 | 12.29 | 7.53 | 6.83 | 2.59 |
|  | 160 | 2.94 | 1.06 | 0.96 | 1.71 |
|  | 800 | 3.36 | 1.24 | 2.09 | -1.12 |
| **Plasma** | 12 | 5.15 | 5.42 | 3.60 | -1.75 |
|  | 160 | 3.70 | 1.87 | 1.97 | 4.23 |
|  | 800 | 5.65 | -1.37 | 3.10 | 1.12 |
| **Muscle** | 12 | 1.98 | 4.94 | 3.86 | 6.66 |
|  | 160 | 3.12 | 5.07 | 3.54 | 6.98 |
|  | 800 | 3.31 | 1.31 | 2.92 | -0.16 |

**Table S3** Method validation of 3,3′′′-binaringenin in rat tissue homogenates (n=5).

| **Sample matrix** | **Con.** | **Intra-day** | | **Inter-day** | |
| --- | --- | --- | --- | --- | --- |
|  | **ng/mL** | **Precision** | **Accuracy** | **Precision** | **Accuracy** |
|  |  | **(RSD, %)** | **(RE, %)** | **(RSD, %)** | **(RE, %)** |
| **Lungs** | 12 | 3.36 | 5.66 | 3.59 | 3.11 |
|  | 160 | 2.17 | 5.58 | 3.39 | 2.94 |
|  | 800 | 1.15 | 4.81 | 0.55 | 3.43 |
| **Liver** | 12 | 4.76 | -5.89 | 4.96 | -2.81 |
|  | 160 | 4.26 | -2.76 | 4.01 | -2.28 |
|  | 800 | 7.87 | -2.88 | 3.24 | -0.18 |
| **Heart** | 12 | 5.17 | -2.35 | 3.05 | 3.17 |
|  | 160 | 3.74 | 3.55 | 3.51 | -2.88 |
|  | 800 | 2.56 | 0.52 | 1.71 | 1.95 |
| **Spleen** | 12 | 2.45 | 1.30 | 1.93 | -1.08 |
|  | 160 | 6.31 | -0.10 | 2.80 | 5.96 |
|  | 800 | 6.13 | 2.85 | 5.12 | -4.41 |
| **Kidney** | 12 | 5.99 | -8.94 | 5.56 | -9.04 |
|  | 160 | 1.25 | -9.20 | 2.40 | -8.24 |
|  | 800 | 2.89 | -4.60 | 2.61 | -5.02 |
| **Brain** | 12 | 6.82 | 0.10 | 3.09 | -1.52 |
|  | 160 | 4.18 | -1.16 | 2.00 | -2.40 |
|  | 800 | 2.17 | 1.79 | 1.95 | 2.01 |
| **Testis** | 12 | 4.41 | 2.92 | 3.79 | 5.44 |
|  | 160 | 5.49 | 4.15 | 3.82 | 0.78 |
|  | 800 | 4.77 | -2.80 | 3.10 | 2.50 |
| **Ovary** | 12 | 7.19 | 4.00 | 4.29 | 1.97 |
|  | 160 | 2.84 | 4.68 | 4.22 | 1.03 |
|  | 800 | 3.10 | 1.10 | 3.31 | 2.93 |
| **Plasma** | 12 | 8.82 | 2.22 | 1.74 | 3.24 |
|  | 160 | 5.15 | -0.73 | 1.30 | 2.05 |
|  | 800 | 6.04 | -1.18 | 2.18 | 2.01 |
| **Muscle** | 12 | 5.32 | 3.64 | 6.70 | 4.66 |
|  | 160 | 3.18 | 4.18 | 3.84 | 2.95 |
|  | 800 | 3.77 | 0.09 | 2.41 | 0.55 |

**Table S4** Method validation of delicaflavone in rat tissue homogenates (n=5).

| **Sample matrix** | **Con.** | **Intra-day** | | **Inter-day** | |
| --- | --- | --- | --- | --- | --- |
|  | **ng/mL** | **Precision** | **Accuracy** | **Precision** | **Accuracy** |
|  |  | **(RSD, %)** | **(RE, %)** | **(RSD, %)** | **(RE, %)** |
| **Lungs** | 12 | 6.01 | 2.75 | 4.78 | 3.83 |
|  | 160 | 5.08 | 3.57 | 2.86 | -2.47 |
|  | 800 | 4.88 | 6.00 | 1.17 | 0.30 |
| **Liver** | 12 | 4.69 | 4.17 | 3.29 | -0.79 |
|  | 160 | 5.74 | 1.67 | 3.01 | -0.45 |
|  | 800 | 2.17 | 0.10 | 1.99 | 0.10 |
| **Heart** | 12 | 3.04 | 1.09 | 2.48 | 4.39 |
|  | 160 | 4.21 | 4.73 | 2.50 | 2.79 |
|  | 800 | 2.17 | 0.17 | 1.95 | 1.28 |
| **Spleen** | 12 | 5.47 | 1.58 | 5.48 | -4.42 |
|  | 160 | 3.73 | 2.37 | 1.32 | 3.62 |
|  | 800 | 2.92 | 1.63 | 2.33 | 2.35 |
| **Kidney** | 12 | 9.14 | -3.72 | 4.12 | 1.26 |
|  | 160 | 6.69 | 2.20 | 3.73 | 3.83 |
|  | 800 | 8.05 | 0.20 | 6.46 | 0.24 |
| **Brain** | 12 | 4.33 | 7.14 | 4.18 | 1.99 |
|  | 160 | 5.21 | -1.94 | 3.36 | 1.49 |
|  | 800 | 4.72 | -0.55 | 3.66 | 3.38 |
| **Testis** | 12 | 10.36 | 1.26 | 6.59 | 10.58 |
|  | 160 | 3.11 | 0.22 | 2.20 | 0.14 |
|  | 800 | 4.15 | -6.17 | 2.72 | -5.75 |
| **Ovary** | 12 | 8.54 | 1.74 | 2.64 | 2.51 |
|  | 160 | 5.37 | -3.74 | 4.86 | -5.49 |
|  | 800 | 3.93 | -0.07 | 3.29 | 0.34 |
| **Plasma** | 12 | 5.62 | 2.28 | 4.02 | 3.62 |
|  | 160 | 6.90 | 1.17 | 1.81 | 2.73 |
|  | 800 | 3.93 | -1.05 | 1.70 | 1.79 |
| **Muscle** | 12 | 4.12 | 1.26 | 4.07 | -0.19 |
|  | 160 | 6.69 | 2.20 | 3.73 | 3.83 |
|  | 800 | 7.07 | -0.55 | 6.46 | 0.24 |

| **Table S5** Matrix effect and extraction recovery of robustflavone in rat tissue homogenates (n=5). | | | | | |
| --- | --- | --- | --- | --- | --- |
| **Sample matrix** | **Spiked con.** | **Matrix effect** | | **Extraction recovery** | |
|  | **(ng/mL)** | **Mean ± SD (%)** | **RSD(%)** | **Mean ± SD (%)** | **RSD(%)** |
| **Lungs** | 12 | 102.10±6.64 | 6.50 | 94.39±9.77 | 10.35 |
|  | 160 | 106.96±3.29 | 3.08 | 100.24±4.31 | 4.29 |
|  | 800 | 104.52±3.20 | 3.06 | 98.62±1.10 | 1.12 |
| **Liver** | 12 | 108.05±4.05 | 3.75 | 95.85±5.41 | 5.64 |
|  | 160 | 102.94±3.11 | 3.02 | 100.03±5.78 | 5.78 |
|  | 800 | 105.41±1.77 | 1.68 | 97.50±0.87 | 0.89 |
| **Heart** | 12 | 94.33±0.17 | 0.18 | 108.22±6.84 | 6.32 |
|  | 160 | 98.69±3.32 | 3.36 | 103.75±1.27 | 1.22 |
|  | 800 | 101.53±0.99 | 0.98 | 100.85±3.72 | 3.69 |
| **Spleen** | 12 | 94.46±3.54 | 3.75 | 91.71±4.85 | 5.29 |
|  | 160 | 101.71±1.89 | 1.86 | 91.43±5.06 | 5.54 |
|  | 800 | 102.50±2.05 | 2.00 | 92.45±4.52 | 4.89 |
| **Kidney** | 12 | 100.13±10.04 | 10.03 | 93.22±0.75 | 0.80 |
|  | 160 | 100.21±4.25 | 4.24 | 90.13±8.92 | 9.90 |
|  | 800 | 100.27±4.38 | 4.37 | 100.17±5.03 | 5.02 |
| **Brain** | 12 | 91.66±1.36 | 1.49 | 93.03±0.97 | 1.04 |
|  | 160 | 100.04±1.38 | 1.38 | 97.48±5.89 | 6.05 |
|  | 800 | 99.24±0.33 | 0.33 | 99.30±0.36 | 0.36 |
| **Testis** | 12 | 99.82±4.04 | 4.05 | 97.55±1.32 | 1.36 |
|  | 160 | 104.69±6.47 | 6.18 | 100.90±5.85 | 5.80 |
|  | 800 | 129.36±12.38 | 9.57 | 79.75±11.59 | 14.53 |
| **Ovary** | 12 | 105.93±1.20 | 1.13 | 80.05±0.15 | 0.19 |
|  | 160 | 105.35±0.51 | 0.48 | 99.34±9.23 | 9.29 |
|  | 800 | 103.75±2.52 | 2.43 | 88.30±4.94 | 5.59 |
| **Plasma** | 12 | 101.37±3.61 | 3.56 | 100.68±1.59 | 0.02 |
|  | 160 | 99.59±4.25 | 4.27 | 100±1.91 | 0.02 |
|  | 800 | 100.27±1.81 | 1.80 | 100.46±3.04 | 0.03 |
| **Muscle** | 12 | 99.40±2.07 | 2.08 | 89.99±0.71 | 0.79 |
|  | 160 | 104.08±0.32 | 0.31 | 100.98±1.50 | 1.49 |
|  | 800 | 108.87±1.04 | 0.95 | 93.50±3.90 | 4.17 |

| **Table S6** Matrix effect and extraction recovery of 2′′,3′′-dihydro-3,3′′′-biapigenin in rat tissue homogenates (n=5). | | | | | |
| --- | --- | --- | --- | --- | --- |
| **Sample matrix** | **Spiked con.** | **Matrix effect** | | **Extraction recovery** | |
|  | **(ng/mL)** | **Mean ± SD (%)** | **RSD(%)** | **Mean ± SD (%)** | **RSD(%)** |
| **Lungs** | 12 | 95.2±2.58 | 2.71 | 100.10±4.46 | 4.46 |
|  | 160 | 108.80±2.50 | 2.30 | 93.98±4.47 | 4.76 |
|  | 800 | 99.37±1.49 | 1.50 | 97.80±1.15 | 1.18 |
| **Liver** | 12 | 90.49±3.39 | 3.74 | 101.76±13.62 | 13.38 |
|  | 160 | 93.35±6.91 | 7.40 | 98.43±2.35 | 2.38 |
|  | 800 | 97.63±1.23 | 1.26 | 92.62±4.64 | 5.01 |
| **Heart** | 12 | 89.25±1.01 | 1.13 | 111.64±0.86 | 0.77 |
|  | 160 | 101.74±4.03 | 3.96 | 100.44±0.22 | 0.22 |
|  | 800 | 97.46±1.50 | 1.54 | 104.17±4.30 | 4.13 |
| **Spleen** | 12 | 95.22±1.18 | 1.24 | 89.92±5.63 | 6.26 |
|  | 160 | 97.99±6.77 | 6.91 | 91.76±7.86 | 8.56 |
|  | 800 | 99.29±2.99 | 3.01 | 85.64±3.00 | 3.50 |
| **Kidney** | 12 | 94.68±5.04 | 5.33 | 93.88±14.85 | 15.81 |
|  | 160 | 89.96±3.97 | 4.41 | 104.53±7.61 | 7.28 |
|  | 800 | 93.27±1.85 | 1.98 | 99.82±3.79 | 3.80 |
| **Brain** | 12 | 88.33±5.76 | 6.52 | 94.47±12.29 | 13.01 |
|  | 160 | 103.28±3.45 | 3.34 | 89.88±7.14 | 7.95 |
|  | 800 | 99.44±3.89 | 3.91 | 96.97±0.52 | 0.53 |
| **Testis** | 12 | 96.98±6.16 | 6.36 | 100.71±0.55 | 0.55 |
|  | 160 | 104.61±0.27 | 0.26 | 94.98±3.12 | 3.28 |
|  | 800 | 105.30±4.45 | 4.23 | 93.23±1.81 | 1.94 |
| **Ovary** | 12 | 97.81±1.93 | 1.97 | 88.88±4.85 | 5.46 |
|  | 160 | 102.33±4.37 | 4.27 | 98.91±4.38 | 4.42 |
|  | 800 | 96.58±1.36 | 1.40 | 97.11±1.14 | 1.17 |
| **Plasma** | 12 | 94.73±3.41 | 3.60 | 96.51±1.62 | 0.02 |
|  | 160 | 98.92±2.18 | 2.21 | 105.06±1.90 | 0.02 |
|  | 800 | 101.18±3.51 | 3.47 | 95.23±0.09 | 0.01 |
| **Muscle** | 12 | 96.95±3.10 | 3.20 | 105.91±1.81 | 1.71 |
|  | 160 | 103.21±0.94 | 0.91 | 102.15±3.17 | 3.10 |
|  | 800 | 100.50±3.91 | 3.89 | 94.89±10.20 | 10.75 |

| **Table S7** Matrix effect and extraction recovery of 3,3′′′-binaringenin in rat tissue homogenates (n=5). | | | | | |
| --- | --- | --- | --- | --- | --- |
| **Sample matrix** | **Spiked con.** | **Matrix effect** | | **Extraction recovery** | |
|  | **(ng/mL)** | **Mean ± SD (%)** | **RSD(%)** | **Mean ± SD (%)** | **RSD(%)** |
| **Lungs** | 12 | 100.94±3.12 | 3.09 | 101.68±1.56 | 1.53 |
|  | 160 | 99.56±0.74 | 0.74 | 96.41±2.43 | 2.52 |
|  | 800 | 107.49±3.50 | 3.25 | 94.98±2.38 | 2.51 |
| **Liver** | 12 | 98.22±11.95 | 12.16 | 100.67±0.14 | 0.14 |
|  | 160 | 91.97±5.92 | 6.44 | 95.05±4.07 | 4.28 |
|  | 800 | 102.50±1.02 | 0.99 | 90.56±12.51 | 13.82 |
| **Heart** | 12 | 87.84±1.23 | 1.40 | 118.76±3.62 | 3.05 |
|  | 160 | 95.82±2.73 | 2.85 | 101.25±1.89 | 1.87 |
|  | 800 | 98.75±1.94 | 1.96 | 102.96±3.11 | 3.02 |
| **Spleen** | 12 | 97.35±0.28 | 0.29 | 99.54±2.55 | 2.57 |
|  | 160 | 99.06±1.33 | 1.34 | 87.21±5.82 | 6.67 |
|  | 800 | 103.84±2.39 | 2.30 | 94.96±6.71 | 7.06 |
| **Kidney** | 12 | 102.73±1.90 | 1.85 | 84.81±0.03 | 0.03 |
|  | 160 | 86.73±1.99 | 2.29 | 101.02±5.15 | 5.09 |
|  | 800 | 92.74±5.19 | 5.60 | 100.96±5.39 | 5.34 |
| **Brain** | 12 | 107.45±10.08 | 9.38 | 85.25±4.87 | 5.71 |
|  | 160 | 98.40±2.57 | 2.61 | 92.99±3.81 | 4.10 |
|  | 800 | 96.24±2.26 | 2.34 | 102.94±0.15 | 0.15 |
| **Testis** | 12 | 104.79±2.25 | 2.15 | 93.63±9.39 | 10.03 |
|  | 160 | 100.61±1.55 | 1.54 | 99.84±1.17 | 1.17 |
|  | 800 | 111.21±1.12 | 1.01 | 91.40±5.81 | 6.36 |
| **Ovary** | 12 | 108.75±0.23 | 0.21 | 90.30±6.44 | 7.14 |
|  | 160 | 95.01±5.15 | 5.42 | 99.58±0.52 | 0.52 |
|  | 800 | 103.09±0.82 | 0.79 | 100.73±3.25 | 3.22 |
| **Plasma** | 12 | 99.07±1.72 | 1.74 | 103.28±7.97 | 0.08 |
|  | 160 | 98.85±1.43 | 1.45 | 97.97±2.94 | 0.03 |
|  | 800 | 100.418±2.45 | 2.44 | 100.19±4.33 | 0.04 |
| **Muscle** | 12 | 89.97±5.48 | 6.09 | 103.25±3.40 | 3.29 |
|  | 160 | 100.50±3.02 | 3.01 | 99.32±2.20 | 2.22 |
|  | 800 | 105.54±1.13 | 1.07 | 90.28±5.21 | 5.77 |

| **Table S8** Matrix effect and extraction recovery of delicaflavone in rat tissue homogenates (n=5). | | | | | |
| --- | --- | --- | --- | --- | --- |
| **Sample matrix** | **Spiked con.** | **Matrix effect** | | **Extraction recovery** | |
|  | **(ng/mL)** | **Mean ± SD (%)** | **RSD(%)** | **Mean ± SD (%)** | **RSD(%)** |
| **Lungs** | 12 | 98.46±3.99 | 4.05 | 98.87±0.27 | 0.27 |
|  | 160 | 96.79±1.73 | 1.79 | 91.69±0.41 | 0.45 |
|  | 800 | 98.73±1.52 | 1.54 | 101.96±2.69 | 2.64 |
| **Liver** | 12 | 94.66±3.66 | 3.87 | 105.93±6.60 | 6.23 |
|  | 160 | 88.40±3.91 | 4.43 | 104.95±5.47 | 5.21 |
|  | 800 | 97.23±1.93 | 1.98 | 102.46±0.77 | 0.75 |
| **Heart** | 12 | 91.66±3.90 | 4.26 | 103.75±3.30 | 3.18 |
|  | 160 | 93.27±1.69 | 1.81 | 103.61±0.27 | 0.26 |
|  | 800 | 99.93±5.10 | 5.10 | 101.90±7.29 | 7.15 |
| **Spleen** | 12 | 101.45±1.79 | 1.77 | 94.26±16.28 | 17.27 |
|  | 160 | 91.85±4.86 | 5.29 | 104.41±3.27 | 3.13 |
|  | 800 | 104.81±6.06 | 5.78 | 94.78±5.61 | 5.92 |
| **Kidney** | 12 | 95.80±1.16 | 1.21 | 90.81±8.09 | 8.91 |
|  | 160 | 89.72±2.34 | 2.61 | 96.42±6.54 | 6.78 |
|  | 800 | 94.89±1.71 | 1.80 | 99.44±1.09 | 1.09 |
| **Brain** | 12 | 102.14±8.13 | 7.96 | 90.88±13.75 | 15.13 |
|  | 160 | 92.00±2.73 | 2.97 | 96.61±5.18 | 5.36 |
|  | 800 | 95.76±1.72 | 1.80 | 99.44±3.05 | 3.07 |
| **Testis** | 12 | 98.13±6.82 | 6.95 | 99.01±1.09 | 1.10 |
|  | 160 | 95.76±5.67 | 5.92 | 95.39±7.20 | 7.55 |
|  | 800 | 102.70±6.37 | 6.21 | 93.36±4.12 | 4.41 |
| **Ovary** | 12 | 93.11±4.57 | 4.90 | 101.64±3.45 | 3.40 |
|  | 160 | 90.63±4.61 | 5.08 | 110.07±0.96 | 0.87 |
|  | 800 | 103.92±4.41 | 4.24 | 98.19±4.62 | 4.71 |
| **Plasma** | 12 | 103.15±4.15 | 4.02 | 92.65±2.50 | 0.03 |
|  | 160 | 101.45±2.06 | 2.03 | 100.00±2.72 | 0.03 |
|  | 800 | 102.81±1.95 | 1.90 | 98.35±2.71 | 0.03 |
| **Muscle** | 12 | 96.71±4.17 | 4.32 | 96.14±11.25 | 11.71 |
|  | 160 | 92.05±0.54 | 0.58 | 104.09±4.95 | 4.76 |
|  | 800 | 97.42±3.65 | 3.75 | 104.98±0.75 | 0.71 |

**Table S9** The stability of robustflavone in rat tissue homogenates (n=5).

| **Sample**  **matrix** | **Spiked**  **con.** | **Bench-top**  **stability** | | **Short-term**  **stability** | | **Freeze-thaw**  **stability** | | **Long-term**  **stability** | |
| --- | --- | --- | --- | --- | --- | --- | --- | --- | --- |
|  |  | **(37℃, 8h)** | | **(4℃, 12 h)** | | **(three cycles)** | | **(-80℃, 60 days)** | |
|  | **(ng/mL)** | **Bias**  **(%)** | **RSD**  **(%)** | **Bias**  **(%)** | **RSD**  **(%)** | **Bias**  **(%)** | **RSD**  **(%)** | **Bias**  **(%)** | **RSD**  **(%)** |
| **Lungs** | 12 | -3.88 | 14.16 | -3.29 | 16.61 | -9.76 | 4.49 | 7.66 | 4.63 |
|  | 160 | 1.25 | 3.09 | 3.55 | 2.90 | -0.65 | 4.63 | 2.13 | 4.34 |
|  | 800 | -3.14 | 3.29 | 2.22 | 2.27 | -2.13 | 2.75 | 3.53 | 4.22 |
| **Liver** | 12 | -5.55 | 3.83 | 3.08 | 3.19 | -8.12 | 8.50 | 8.83 | 1.45 |
|  | 160 | -1.04 | 4.10 | -0.47 | 2.15 | -3.01 | 4.11 | 2.44 | 2.18 |
|  | 800 | -1.12 | 1.50 | -1.51 | 1.83 | 0.57 | 1.20 | 2.33 | 2.27 |
| **Heart** | 12 | -2.93 | 6.44 | 3.89 | 5.87 | -3.79 | 7.31 | -1.02 | 5.00 |
|  | 160 | 2.14 | 3.54 | 0.65 | 3.48 | 2.27 | 2.90 | -2.68 | 5.97 |
|  | 800 | 1.90 | 4.66 | 1.35 | 3.35 | -1.33 | 1.81 | -1.22 | 5.04 |
| **Spleen** | 12 | 0.64 | 2.73 | -1.33 | 7.50 | 6.57 | 12.06 | 3.31 | 4.46 |
|  | 160 | -3.41 | 3.36 | 2.51 | 3.39 | 1.47 | 1.46 | 3.43 | 3.48 |
|  | 800 | -5.42 | 5.65 | -3.42 | 3.22 | 3.21 | 3.08 | 5.80 | 1.56 |
| **Kidney** | 12 | 6.65 | 4.66 | 7.99 | 5.46 | 1.53 | 5.96 | 3.75 | 7.44 |
|  | 160 | 5.24 | 2.51 | 0.84 | 4.11 | 1.94 | 6.80 | 2.99 | 3.37 |
|  | 800 | 1.19 | 1.16 | 0.22 | 2.90 | 0.73 | 2.33 | 4.16 | 2.01 |
| **Brain** | 12 | 2.14 | 5.74 | -1.78 | 2.95 | 10.10 | 1.86 | 3.63 | 9.95 |
|  | 160 | 5.51 | 2.36 | 2.00 | 2.05 | 10.14 | 2.20 | 4.51 | 3.80 |
|  | 800 | 5.23 | 2.79 | -0.45 | 2.39 | 7.99 | 2.39 | -0.05 | 3.66 |
| **Testis** | 12 | 0.98 | 5.27 | -0.67 | 4.02 | 0.00 | 8.09 | 3.05 | 3.33 |
|  | 160 | 1.58 | 1.70 | 1.59 | 3.27 | 0.08 | 1.29 | 3.27 | 1.02 |
|  | 800 | 1.04 | 2.65 | -0.12 | 1.78 | 2.49 | 2.49 | 3.58 | 1.66 |
| **Ovary** | 12 | -3.63 | 6.51 | 1.67 | 4.43 | 2.81 | 4.98 | -2.11 | 8.24 |
|  | 160 | 3.35 | 2.23 | -1.75 | 8.30 | 3.90 | 1.39 | 6.39 | 4.15 |
|  | 800 | 5.33 | 4.90 | 3.60 | 2.83 | 1.40 | 5.46 | -4.91 | 8.31 |
| **Plasma** | 12 | 5.48 | 5.12 | 4.85 | 10.47 | -1.17 | 7.47 | -2.51 | 6.56 |
|  | 160 | 0.17 | 5.62 | 5.69 | 1.82 | 2.54 | 4.32 | 0.10 | 2.06 |
|  | 800 | 1.38 | 3.21 | 0.59 | 6.43 | 3.24 | 1.85 | -3.23 | 2.39 |
| **Muscle** | 12 | 1.31 | 12.23 | 7.47 | 7.68 | -0.42 | 3.07 | -4.00 | 13.90 |
|  | 160 | -5.42 | 5.62 | -8.28 | 5.97 | 4.01 | 3.86 | 0.35 | 8.73 |
|  | 800 | 4.48 | 2.54 | 0.78 | 6.72 | 1.32 | 1.11 | -1.54 | 7.69 |

**Table S10** The stability of 2′′,3′′-dihydro-3,3′′′-biapigenin in rat tissue homogenates (n=5).

| **Sample**  **matrix** | **Spiked**  **con.** | **Bench-top**  **stability** | | **Short-term**  **stability** | | **Freeze-thaw**  **stability** | | **Long-term**  **stability** | |
| --- | --- | --- | --- | --- | --- | --- | --- | --- | --- |
|  |  | **(37℃, 8h)** | | **(4℃, 12 h)** | | **(three cycles)** | | **(-80℃, 60 days)** | |
|  | **(ng/mL)** | **Bias**  **(%)** | **RSD**  **(%)** | **Bias**  **(%)** | **RSD**  **(%)** | **Bias**  **(%)** | **RSD**  **(%)** | **Bias**  **(%)** | **RSD**  **(%)** |
| **Lungs** | 12 | -3.63 | 7.63 | -0.71 | 5.13 | -3.52 | 6.24 | 8.28 | 2.31 |
|  | 160 | -2.04 | 5.61 | 0.26 | 5.44 | -8.38 | 5.55 | 1.92 | 3.78 |
|  | 800 | -6.69 | 10.15 | -2.57 | 3.11 | -3.19 | 4.60 | 5.07 | 4.22 |
| **Liver** | 12 | -3.74 | 3.02 | -5.35 | 6.96 | -8.63 | 0.72 | 4.64 | 3.14 |
|  | 160 | -1.30 | 4.60 | -3.89 | 4.27 | -7.81 | 1.09 | 4.42 | 3.14 |
|  | 800 | 0.39 | 1.28 | 0.12 | 2.55 | -2.74 | 2.23 | 1.11 | 1.69 |
| **Heart** | 12 | 6.26 | 10.10 | 8.72 | 3.59 | -0.77 | 5.91 | -0.13 | 3.37 |
|  | 160 | 4.55 | 1.32 | 0.39 | 2.70 | 1.80 | 2.61 | -6.61 | 5.74 |
|  | 800 | 0.07 | 2.53 | 2.76 | 1.27 | -1.26 | 2.33 | -0.13 | 5.77 |
| **Spleen** | 12 | -11.27 | 2.27 | -9.95 | 7.96 | 1.81 | 4.18 | 4.27 | 2.14 |
|  | 160 | -11.09 | 3.17 | 3.45 | 3.14 | 3.09 | 1.24 | 4.45 | 1.52 |
|  | 800 | -10.21 | 5.06 | -8.80 | 3.90 | 3.04 | 2.55 | 5.68 | 4.52 |
| **Kidney** | 12 | 10.50 | 3.92 | 8.14 | 6.70 | 0.39 | 9.11 | -5.88 | 2.11 |
|  | 160 | 4.17 | 3.57 | 2.79 | 2.99 | -2.63 | 3.57 | 0.67 | 1.96 |
|  | 800 | 4.10 | 5.80 | 4.18 | 3.50 | 0.51 | 2.66 | 1.77 | 1.43 |
| **Brain** | 12 | 5.07 | 4.75 | -1.91 | 5.50 | 3.37 | 4.61 | 0.70 | 5.06 |
|  | 160 | 2.35 | 1.57 | -2.56 | 3.53 | 3.42 | 2.48 | 2.48 | 3.79 |
|  | 800 | 2.55 | 3.17 | -2.81 | 4.90 | 3.28 | 3.63 | -0.53 | 5.71 |
| **Testis** | 12 | 1.44 | 5.19 | -3.23 | 5.45 | 7.43 | 4.36 | 2.19 | 6.32 |
|  | 160 | 7.51 | 5.84 | 7.31 | 3.06 | 4.68 | 1.93 | 6.84 | 2.02 |
|  | 800 | 0.49 | 5.73 | 5.45 | 2.73 | 4.78 | 4.33 | 4.98 | 1.52 |
| **Ovary** | 12 | -2.53 | 10.67 | -6.70 | 5.04 | 1.84 | 4.89 | 0.54 | 3.03 |
|  | 160 | -2.47 | 5.81 | 1.21 | 1.43 | 1.78 | 2.42 | 3.92 | 4.14 |
|  | 800 | 2.25 | 1.77 | 0.82 | 3.36 | 2.76 | 1.44 | 1.64 | 2.64 |
| **Plasma** | 12 | 1.27 | 2.89 | 0.59 | 9.88 | -1.22 | 9.54 | -1.42 | 9.17 |
|  | 160 | 0.59 | 4.46 | 4.12 | 2.06 | -1.22 | 7.08 | -2.67 | 3.19 |
|  | 800 | 2.42 | 3.52 | 0.15 | 2.60 | 2.37 | 4.96 | 2.81 | 3.93 |
| **Muscle** | 12 | -1.02 | 11.55 | 2.79 | 4.68 | 3.61 | 2.37 | -2.32 | 8.04 |
|  | 160 | -3.95 | 3.95 | -9.01 | 4.96 | -1.01 | 2.91 | 2.28 | 5.52 |
|  | 800 | 3.78 | 1.14 | 1.64 | 5.54 | 1.31 | 1.78 | -1.97 | 7.27 |

**Table S11** The stability of 3,3′′′-binaringenin in rat tissue homogenates (n=5).

| **Sample**  **matrix** | **Spiked**  **con.** | **Bench-top**  **stability** | | **Short-term**  **stability** | | **Freeze-thaw**  **stability** | | **Long-term**  **stability** | |
| --- | --- | --- | --- | --- | --- | --- | --- | --- | --- |
|  |  | **(37℃, 8h)** | | **(4℃, 12 h)** | | **(three cycles)** | | **(-80℃, 60 days)** | |
|  | **(ng/mL)** | **Bias**  **(%)** | **RSD**  **(%)** | **Bias**  **(%)** | **RSD**  **(%)** | **Bias**  **(%)** | **RSD**  **(%)** | **Bias**  **(%)** | **RSD**  **(%)** |
| **Lungs** | 12 | -0.93 | 6.24 | 2.99 | 12.63 | -0.32 | 4.98 | 1.96 | 4.91 |
|  | 160 | 1.78 | 3.46 | -1.40 | 4.45 | -2.24 | 4.47 | 1.72 | 5.12 |
|  | 800 | -1.22 | 5.65 | -4.16 | 1.46 | 0.72 | 5.00 | 0.98 | 2.65 |
| **Liver** | 12 | -6.89 | 7.71 | 0.81 | 4.38 | -3.10 | 9.66 | 6.40 | 3.12 |
|  | 160 | -2.46 | 2.07 | -0.08 | 4.99 | -0.63 | 0.87 | 6.12 | 2.85 |
|  | 800 | 0.78 | 0.83 | 2.71 | 2.43 | 1.60 | 1.78 | 4.10 | 4.02 |
| **Heart** | 12 | 0.63 | 3.99 | -0.11 | 6.12 | -12.33 | 5.19 | 3.66 | 4.08 |
|  | 160 | 4.81 | 2.21 | 1.01 | 4.34 | 1.14 | 3.15 | -3.83 | 3.84 |
|  | 800 | 1.53 | 4.40 | 4.22 | 2.46 | 1.62 | 2.00 | -2.25 | 3.65 |
| **Spleen** | 12 | -3.91 | 6.48 | -4.02 | 5.46 | 3.31 | 2.73 | 6.37 | 5.52 |
|  | 160 | -6.06 | 3.85 | 3.96 | 4.30 | 7.90 | 3.91 | 0.53 | 3.03 |
|  | 800 | -5.57 | 4.35 | -5.99 | 3.99 | 2.38 | 2.42 | 4.37 | 3.53 |
| **Kidney** | 12 | 2.75 | 5.53 | 4.72 | 2.59 | 8.89 | 5.93 | -0.68 | 7.73 |
|  | 160 | 3.72 | 4.19 | 6.41 | 2.21 | -0.64 | 1.44 | -1.80 | 1.47 |
|  | 800 | 4.12 | 5.29 | 2.08 | 3.10 | 0.85 | 1.74 | 0.39 | 0.94 |
| **Brain** | 12 | 5.78 | 4.55 | -2.16 | 6.29 | 4.27 | 4.54 | 2.71 | 4.02 |
|  | 160 | 5.77 | 1.72 | -2.56 | 4.62 | 7.58 | 2.90 | -0.10 | 3.48 |
|  | 800 | 4.66 | 1.71 | 2.53 | 2.68 | 6.24 | 2.63 | -1.94 | 4.85 |
| **Testis** | 12 | 9.10 | 5.75 | -3.01 | 8.59 | 6.35 | 4.12 | 5.38 | 2.98 |
|  | 160 | 4.59 | 2.66 | 4.76 | 1.81 | 1.40 | 4.12 | 5.32 | 3.03 |
|  | 800 | 4.74 | 3.73 | 3.27 | 2.72 | 6.30 | 2.68 | 1.96 | 3.00 |
| **Ovary** | 12 | -1.58 | 8.18 | 4.92 | 6.07 | -2.43 | 11.36 | 4.20 | 6.36 |
|  | 160 | -4.02 | 5.66 | -1.21 | 5.41 | 6.61 | 3.54 | -1.68 | 3.70 |
|  | 800 | 4.14 | 3.39 | -4.43 | 4.14 | 2.86 | 4.06 | -5.72 | 6.53 |
| **Plasma** | 12 | 2.44 | 4.97 | 3.91 | 9.69 | 1.52 | 5.32 | -1.29 | 7.92 |
|  | 160 | 1.86 | 2.99 | 4.10 | 2.85 | -0.47 | 3.19 | 1.54 | 2.52 |
|  | 800 | 0.34 | 4.12 | 2.16 | 3.78 | 0.71 | 6.30 | -0.04 | 6.52 |
| **Muscle** | 12 | 5.09 | 2.69 | -2.05 | 10.87 | 2.44 | 6.57 | 5.35 | 5.79 |
|  | 160 | -6.21 | 4.98 | 0.05 | 5.57 | 3.68 | 3.81 | 3.80 | 3.75 |
|  | 800 | 3.43 | 3.31 | -0.61 | 5.76 | -3.60 | 8.59 | -4.08 | 5.17 |

**Table S12** The stability of delicaflavone in rat tissue homogenates (n=5).

| **Sample**  **matrix** | **Spiked**  **con.** | **Bench-top**  **stability** | | **Short-term**  **stability** | | **Freeze-thaw**  **stability** | | **Long-term**  **stability** | |
| --- | --- | --- | --- | --- | --- | --- | --- | --- | --- |
|  |  | **(37℃, 8h)** | | **(4℃, 12 h)** | | **(three cycles)** | | **(-80℃, 60 days)** | |
|  | **(ng/mL)** | **Bias**  **(%)** | **RSD**  **(%)** | **Bias**  **(%)** | **RSD**  **(%)** | **Bias**  **(%)** | **RSD**  **(%)** | **Bias**  **(%)** | **RSD**  **(%)** |
| **Lungs** | 12 | -2.46 | 10.44 | -8.82 | 5.87 | -5.99 | 8.56 | 3.51 | 6.66 |
|  | 160 | 4.61 | 4.00 | -2.65 | 4.85 | 1.09 | 3.18 | 7.67 | 2.33 |
|  | 800 | 2.23 | 1.77 | -7.91 | 3.30 | 0.79 | 6.05 | 1.96 | 2.75 |
| **Liver** | 12 | 1.70 | 4.79 | -2.18 | 6.35 | 4.55 | 7.08 | 4.80 | 5.02 |
|  | 160 | 1.07 | 2.13 | -1.33 | 4.21 | -0.16 | 2.44 | 3.19 | 3.74 |
|  | 800 | 2.25 | 4.03 | 3.14 | 2.37 | 2.74 | 3.93 | 5.14 | 2.94 |
| **Heart** | 12 | 6.10 | 2.97 | 5.03 | 5.94 | -5.01 | 9.84 | 1.68 | 8.06 |
|  | 160 | 0.12 | 1.63 | -3.43 | 5.55 | 0.56 | 1.41 | 8.07 | 3.15 |
|  | 800 | -1.50 | 3.31 | 1.21 | 2.40 | -1.04 | 1.01 | 1.69 | 2.85 |
| **Spleen** | 12 | 7.96 | 7.98 | 11.78 | 6.45 | -9.39 | 16.36 | 5.03 | 7.52 |
|  | 160 | -0.78 | 7.34 | -0.35 | 2.55 | -6.65 | 4.89 | -1.85 | 5.04 |
|  | 800 | 6.58 | 9.65 | 0.94 | 4.91 | -1.46 | 4.00 | -1.25 | 4.47 |
| **Kidney** | 12 | 4.46 | 11.83 | 8.74 | 10.09 | 1.93 | 7.84 | 5.66 | 6.50 |
|  | 160 | -2.01 | 2.76 | -0.06 | 4.63 | -1.63 | 5.35 | -2.45 | 5.32 |
|  | 800 | 2.49 | 1.99 | 2.69 | 1.22 | 4.36 | 1.47 | -0.05 | 2.04 |
| **Brain** | 12 | 3.49 | 4.39 | 1.28 | 6.86 | -3.70 | 7.45 | -3.64 | 6.06 |
|  | 160 | 2.33 | 3.46 | -1.69 | 2.27 | -0.88 | 4.44 | 0.47 | 3.25 |
|  | 800 | 5.23 | 2.68 | 0.31 | 5.74 | 0.16 | 8.69 | -1.65 | 2.30 |
| **Testis** | 12 | -4.62 | 2.89 | -3.30 | 8.35 | -0.82 | 2.22 | -0.37 | 6.02 |
|  | 160 | 1.71 | 3.07 | 0.64 | 4.75 | -8.89 | 4.59 | -1.22 | 4.11 |
|  | 800 | -9.39 | 3.51 | -11.44 | 1.64 | -7.45 | 3.06 | -5.53 | 1.39 |
| **Ovary** | 12 | 6.75 | 1.45 | 4.11 | 5.59 | 1.07 | 10.59 | -3.54 | 5.75 |
|  | 160 | -0.90 | 3.32 | 0.03 | 3.04 | 5.42 | 4.16 | 2.88 | 6.93 |
|  | 800 | 4.36 | 2.27 | -6.16 | 7.37 | 5.82 | 5.79 | 1.70 | 1.99 |
| **Plasma** | 12 | -5.18 | 5.17 | -0.34 | 5.63 | -1.65 | 7.01 | -2.24 | 6.66 |
|  | 160 | -2.15 | 5.40 | 1.89 | 3.89 | 3.18 | 5.57 | -2.37 | 8.81 |
|  | 800 | -1.91 | 6.81 | 3.90 | 4.32 | 4.69 | 5.05 | -0.24 | 2.22 |
| **Muscle** | 12 | 0.67 | 9.42 | -4.85 | 7.83 | -2.98 | 3.69 | -7.83 | 7.90 |
|  | 160 | -1.23 | 7.08 | 2.23 | 2.90 | -1.17 | 4.48 | 2.11 | 1.83 |
|  | 800 | -2.33 | 3.21 | -1.78 | 2.71 | -2.98 | 7.58 | -2.81 | 4.34 |
